# Bitter Taste Receptor TAS2R43 Co-Regulates Mechanisms of Gastric Acid Secretion and Zinc Homeostasis

**DOI:** 10.3390/ijms26136017

**Published:** 2025-06-23

**Authors:** H. Noreen Orth, Philip Pirkwieser, Julia Benthin, Melanie Koehler, Sonja Sterneder, Etkin Parlar, Erika Schaudy, Jory Lietard, Timm Michel, Valerie Boger, Andreas Dunkel, Mark M. Somoza, Veronika Somoza

**Affiliations:** 1 Graduate School of Life Sciences, Technical University of Munich, 85354 Freising, Germany; 2 Leibniz-Institute for Food Systems Biology, Technical University of Munich, 85354 Freising, Germanym.koehler.leibniz-lsb@tum.de (M.K.); a.dunkel.leibniz-lsb@tum.de (A.D.); m.somoza.leibniz-lsb@tum.de (M.M.S.); 3 TUM Junior Fellow, School of Life Sciences, Technical University of Munich, 85354 Freising, Germany; 4 Institute of Physiological Chemistry, Faculty of Chemistry, University of Vienna, 1090 Vienna, Austria; 5 Vienna Doctoral School in Chemistry (DoSChem), University of Vienna, 1090 Vienna, Austria; 6 Department of Inorganic Chemistry, Faculty of Chemistry, University of Vienna, 1090 Vienna, Austria; 7 Chair of Food Chemistry and Molecular Sensory Science, School of Life Sciences, Technical University of Munich, 85354 Freising, Germany; 8 Chair of Nutritional Systems Biology, School of Life Sciences Weihenstephan, Technical University of Munich, 85354 Freising, Germany

**Keywords:** bitter taste receptors, TAS2Rs, zinc, gastric acid secretion, parietal cells, HGT-1 cells, atomic force microscopy

## Abstract

The essential micronutrient zinc is known to inhibit gastric acid secretion (GAS), where its homeostasis is strictly regulated. We hypothesized that the gastric bitter taste receptors, TAS2Rs, regulate the following: (i) zinc-modulated proton secretory activity (PSA) as a key mechanism of GAS and (ii) zinc homeostasis in immortalized parietal cells. To confirm this hypothesis, human gastric tumor cells (HGT-1) were exposed to 100–1000 µM of zinc salts for 30 min in order to quantitate their TAS2R-dependent PSA and intracellular zinc concentration using a fluorescence-based pH sensor and ICP-MS, respectively. Thereby, we identified TAS2R43 as a key player in parietal cell PSA and zinc homeostasis, with both conclusions being verified by a CRISPR-Cas9 knockout approach. Moreover, by regulating the zinc importer protein ZIP14, TAS2R43 proved to perform a protective role against excessive zinc accumulation in immortalized parietal cells.

## 1. Introduction

Bitter taste sensing receptors (TAS2Rs) belong with 25 members to the second largest group of chemosensory G-protein-coupled receptors (GPCRs), with TAS2R2 being the 26th member, as recently described by Lang et al. [1]. TAS2Rs on the tongue are responsible for bitter taste perception. TAS2Rs are transmembrane receptors that are expressed not only in taste bud cells on the tongue, but also extra-orally, such as in parietal cells of the stomach [2]. This distribution has led to the hypothesis that TAS2Rs fulfill functional roles beyond taste perception. In response to bitter-tasting toxic compounds finding their way into the stomach, regardless of oral recognition, TAS2Rs are assumed to initiate, as an act of digestion and defense, cellular pathways that stimulate gastric acid secretion (GAS). Thereby, they aid in the degradation or removal of potential toxins. While the activation of TAS2Rs in taste cells results in a signaling cascade leading to cell depolarization and neurotransmitter release [3,4], their activation in parietal cells induces proton secretion, a key component of GAS [2]. Specifically, functional involvement in proton secretory activity (PSA), as key mechanism of GAS, was demonstrated in immortalized human parietal cell systems for the individual bitter taste receptors TAS2R1, 4, 16, 38, and 43, and was verified by siRNA knockdown (kd) or CRISPR-Cas9 knockout (ko) approaches [2,5,6,7]. Recent evidence also suggests that TAS2Rs are involved in the cellular uptake of small molecules [8] and the cellular sensing of divalent metal salts such as MgCl_2_, MnCl_2_, and FeSO_4_ [9,10].

The divalent metal zinc, a prominent trace element in foods, is well known for its importance in immune functions, cellular homeostasis, and membrane integrity [11,12,13]. On a cellular level, zinc is described to induce several signaling cascades, and has been hypothesized to function as a novel intracellular second messenger and neurotransmitter [14]. The cellular homeostasis of zinc is strictly controlled by the two key zinc transporter families, Zrt/Irt-like proteins (ZIPs) and Zn-transporters (ZnTs). The ZnT family is responsible for the transport of zinc out of the cell into intracellular compartments, which thereby decreases zinc levels in the cytoplasm. Proteins from the ZIP family are responsible for raising the zinc concentration in the cytoplasm [15], of which ZIP14 is chiefly responsible for maintaining the barrier function in the intestinal tract [16]. Zinc is presumed not to be membrane permeable, even though the literature on its absorption is scarce [17]. Within the intestinal tract, the uptake of zinc from foods has been described to take place in the small intestine [18]. In vitro studies on the uptake of zinc and its biological functions are therefore often executed on Caco-2 cells [19,20,21], although the absorption of zinc in the stomach cannot be ruled out, as it was previously shown for rabbit parietal cells [22].

The National Institutes of Health recommend a daily zinc intake of 8–11 mg (Dietary Reference Intakes, USA, status 2022 [23]), recognizing its importance as an essential trace element in metabolism. To date, no toxicity has been ascertained for naturally occurring zinc, although a dosage of 50 mg in supplement form may cause gastrointestinal distress [24].

In foods, zinc may act as a potent taste modulator, decreasing bitter and sweet taste perception or altering taste preferences for salt and bitter qualities [25,26,27]. At higher doses of 300 µM, dietary ingested zinc salts, ZnCl_2_ and ZnSO_4_, were demonstrated to inhibit GAS, although no underlying mechanism has been shown yet [28,29,30]. Building on our previous research on TAS2Rs regulating mechanisms of GAS [2,5,6,7], our primary hypothesis was that zinc acts on parietal PSA as a key mechanism of GAS via TAS2Rs. Since TAS2Rs have been demonstrated to also regulate the cellular uptake of small molecules [8], our secondary hypothesis was that TAS2Rs are involved in mechanisms regulating the cellular uptake of zinc. As a consequence, TAS2Rs help to protect the parietal cell from excessive zinc accumulation.

Our work utilized the human gastric tumor cell line (HGT-1), as it is a suitable model to assess the impact of zinc on the functional role of TAS2Rs as transmembrane proteins regulating the PSA and mechanisms of zinc homeostasis in parietal cells of the stomach. As a result, TAS2R43 was identified as a receptor that is targeted by zinc, as it demonstrated (i) a decreased PSA in homozygous TAS2R43 CRISPR-Cas-9ko cells and (ii) higher intracellular zinc concentrations compared to wild type cells. Mechanistically, this difference in intracellular zinc concentration was demonstrated to result from changes in the cells’ membrane stiffness in the absence of TAS2R43 and increased expression of the zinc transporter protein ZIP14, which were observed by means of atomic force microscopy (AFM) and immunohistochemistry, respectively.

## 2. Results

Our primary hypothesis was that TAS2Rs regulate PSA as a response to zinc exposure as a key mechanism of GAS. Secondary, we hypothesized the involvement of TAS2Rs in cellular zinc homeostasis.

### 2.1. Zinc-Induced Reduction of Proton Secretory Activity (PSA) in HGT-1 Cells Is Mediated by TAS2Rs

**Figure 1:**
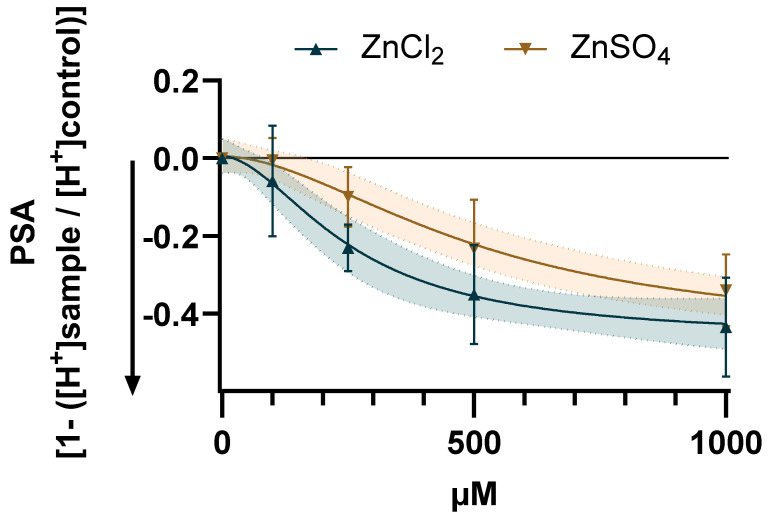
PSA, calculated as ratio of proton concentration in treated vs. control cells subtracted from 1, in HGT-1 cells after exposure to ZnCl_2_ and ZnSO_4_ (0–1000 µM) as mean ± SD with nonlinear fit curve and its confidence interval (95%). Data (*n* = 4–6, t.r. = 3) are displayed as mean ± SD.

In accordance with previous reports expounding that zinc inhibits mechanisms of gastric acid secretion [28,29,30], the PSA was studied in HGT-1 cells to address our primary hypothesis. The PSA assay conducted by means of SNARF-1AM fluorescent dye was performed with ZnCl_2_ and ZnSO_4_ as well as further metal salts with sulphate or chloride anions (MgSO_4_, Na_2_SO_4_, KCl, and NaCl) to exclude potential anion attribution to the PSA. The PSA responded predominantly to the zinc ion and not to the other tested cations (Figure S1A). There was no significant difference between the treatment with ZnCl_2_ and that with ZnSO_4_, and further experiments were therefore conducted with ZnCl_2_. Whereas the results of the treatment with 100 µM ZnCl_2_ did not differ from those of non-treated controls, a decreased PSA value of 0.23 ± 0.06 resulted from the treatment with 250 µM ZnCl_2_ and decreased to −0.35 ± 0.13 and −0.44 ± 0.13 with 500 µM and 1000 µM ZnCl_2_, respectively (Figure 1).

**Figure 2:**
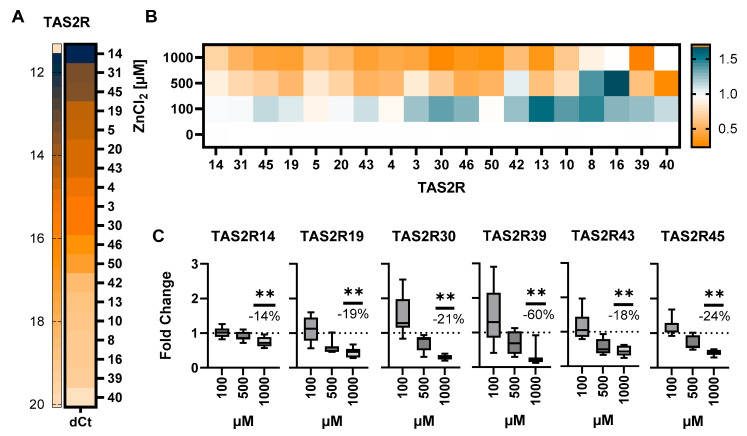
(**A**) Heatmap scaling the relative expression of TAS2Rs as dCt in untreated HGT-1 cells, excluding receptors expressed with a Ct value ≥ 38. (**B**) Heatmap displaying the mean change in gene expression of 21 bitter taste receptors (exclusion of TAS2Rs with Ct ≥ 38 [2]) in HGT-1 cells due to 30 min incubation with 100, 500, and 1000 µM ZnCl_2_. Upregulation is expressed in orange and downregulation in blue, with control = 1 set as white. The fold change is displayed as geometric mean, *n* = 4–6, t.r. = 3. (**C**) Change in concentration-dependent mRNA expression as FC of bitter taste receptors TAS2R 14, 19, 30, 39, 43, and 45 in HGT-1 cells after treatment with ZnCl_2_ for 100, 500, and 1000 µM compared to control cells, which were treated only with medium (fold change = 1.0). Data shown as mean ± SEM, percentage change respective to untreated control cells, *n* = 4–6, t.r. = 3, statistics: Holm-Sídák *t*-test method, significant differences to control expressed with ** = *p* ≤ 0.01.

Building on previous results, demonstrating PSA to depend on the functional involvement of TAS2Rs with respective changes in mRNA levels [5,31], HGT-1 cells treated with 100, 500, and 1000 µM ZnCl_2_ for 30 min were subjected to a TAS2R mRNA screening by RT-qPCR analysis (Figure 2A–C).

**Figure 3:**
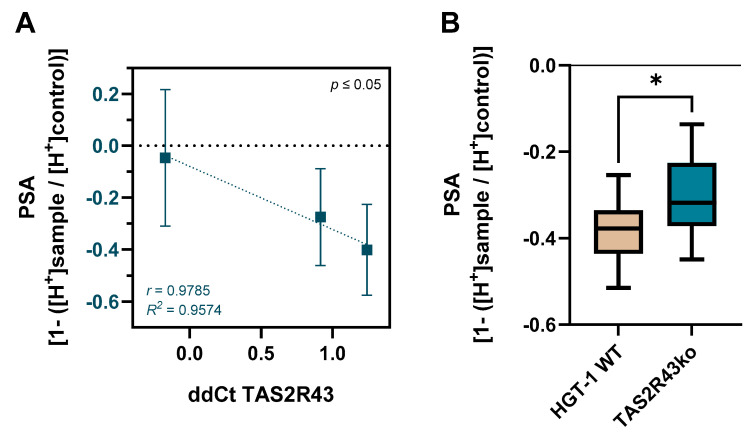
(**A**) Correlation of the ddCt values of TAS2R43 with the PSA (r = 0.9785, *p* ≤ 0.05) for HGT-1 cells treated for 30 min with 1000 µM ZnCl_2_. After a successful Shapiro–Wilk test proving normality, the Pearson correlation test was conducted and resulted in significant correlations. Data with *n* = 4–6, t.r. = 3 are displayed as mean ± SD. (**B**) Boxplot comparing the effect of 1000 µM zinc salt on HGT-1 WT to TAS2R43ko cells. The PSA was reduced in TAS2R43ko cells (0.36 ± 0.11) by 34% compared to the wild type cells (0.46 ± 0.08) after treatment with 1000 µM ZnCl_2_, but no difference was found for TAS2R39ko cells (0.59 ± 0.4). Statistical analyses were performed with Holm-Sídák *t*-test method (*n* = 4–6, t.r. = 3) and significance displayed with * = *p* ≤ 0.05.

In the first place, the mRNA screening revealed that most TAS2Rs are well expressed in HGT-1 cells (Figure 2A), with Ct values ≥ 38 [2]. Figure 2B shows that, in comparison to untreated control cells (fold change (FC) = 1), the exposure to zinc lead to a downregulation (FC < 1), with a changed expression (*p* < 0.01) of individual TAS2R genes being observed upon treatment with 1000 µM (Figure 2C). Among these receptors, TAS2R39 responded despite its comparably low baseline expression with the lowest FC (0.24 ± 0.32; −60% decrease vs. non-treated controls; *p* < 0.01), followed by TAS2R30 (0.29 ± 0.06; −21% decrease vs. non-treated controls; *p* < 0.01) and TAS2R43 (0.44 ± 0.13; −18% decrease vs. non-treated controls; *p* < 0.01). However, TAS2R43 was, according to its dCt value of 14.59, highly expressed, compared to other TAS2Rs responding to zinc (Figure 2A). Pearson tests revealed the ddCt of TAS2R43 as the only receptor to have a correlation with the PSA (r = 0.9785, *p* ≤ 0.05) (Figure 3A, Table S1). Therefore, further experiments addressing the inhibiting involvement of TAS2R in mechanisms of PSA focused on TAS2R43, as well as on TAS2R39, which responded despite its comparatively low expression in HGT-1 cells (dCt = 19.17), with a strong effect size (Table S2, Figure S1B,C). A homozygote CRISPR-Cas9ko of TAS2R39, verified through the alignment of Sanger sequencing data, was conducted in HGT-1 cells (Figure S2). By means of CRISPR-Cas9 TAS2R39ko and 43ko cells, providing a stable and more specific alternative to siRNA knockdown approaches, PSA upon zinc exposure was validated. TAS2R60, 41, 7, 9, 38, and 1 were excluded from the experiments due to their Ct values over 38 (Figure 2A) [2].

The PSA in TAS2R43ko cells was higher than that in HGT-1 WT cells, reaching statistical significance when treated with 1000 µM ZnCl_2_, as the following PSA values of the TAS2R43ko cells indicate: 0.01 ± 0.14, −0.10 ± 0.26, −0.23 ± 0.27 for 100, 500, and 1000 µM ZnCl_2_, respectively (Figure 3B). Since no difference was observed for TAS2R39ko vs. WT cells (Figure S1B), the focus of the subsequent experiments was shifted to TAS2R43.

An important aspect of GAS is the mobilization of Ca^2+^ through the modulation of the activity of acid-secreting H^+^-K^+^-ATPase in parietal cells [32], while being also an important factor in the downstream signaling cascade upon TAS2R activation [33,34]. The calcium mobilization was therefore measured using Cal-520 in WT and TAS2R43ko cells. As displayed in Figure S3A–C, the curves in HGT-1 WT cells instantly proceed upon the addition of zinc after 60 s into the negative range, which could result from an immediate cellular release of Ca^2+^ into the extracellular compartment. The curve of TAS2R43ko cells deviate above the WT signals, which could, despite a possible reaction of zinc with the dye, speak for an increased cellular calcium release in TAS2R43ko cells. With exposure to higher concentrations of zinc, generally, the release of more calcium was indicated.

### 2.2. Protective Role of TAS2R43 Against Excessive Cellular Zn^2+^ Uptake

**Figure 4:**
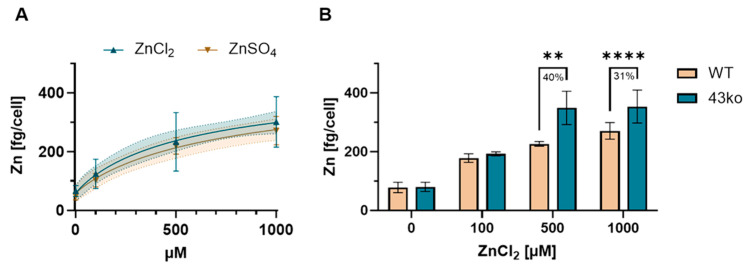
(**A**) Measured uptake of Zn^2+^ in fg/cell after treatment with ZnCl_2_ (0, 100, 500, and 1000 µM) displayed as mean ± SD with *n* = 3–4, t.r. = 3–5. (**B**) Amount of Zn^2+^ in WT (77.78, 178.14, 226.66, 270.69) and 43ko (79.80, 192.38, 348.68, 353.43) cells, displayed with ±SD, *n* = 3–4, t.r. = 3–5. Statistics obtained by two-way ANOVA. Significance displayed with ** = *p* ≤ 0.01, **** = *p* ≤ 0.0001.

TAS2Rs were discovered to contribute to the uptake of small molecules [8], raising the assumption that they also contribute to zinc homeostasis, which is addressed by our secondary hypothesis. To measure the concentration of zinc in parietal cells, the Zn^2+^-levels in HGT-1 cells were quantitated by means of ICP-MS in a bulk experiment. Figure 4A provides the obtained experimental data on the quantitated amount of Zn^2+^ in the cells after exposure to the two zinc salts ZnCl_2_ and ZnSO_4_ (0 = untreated, 100, 500, and 1000 µM). The intracellular zinc concentration increased dose-dependently for 0, 100, 500, and 1000 µM as follows: 43.1 ± 12.5, 124 ± 49.6, 233 ± 99.4, and 301 ± 86.2 fg/cell for ZnCl_2_, and 66 ± 18.6, 96.6 ± 27.3, 223 ± 34.9, and 288 ± 52.0 fg/cell for ZnSO_4_, respectively. Compared to control cells, percentage increases of 254 ± 43% and 358 ± 29% for ZnCl_2_, and 417 ± 16% and 569 ± 18% for ZnSO_4_, when treated with 500 and 1000 µM, respectively. Notably, the anion of the zinc salts did not impact the cellular zinc concentration, as the results of the two salts did not differ (Figure 4A). Referring back to their mRNA expression and their significantly reduced fold change upon treatment with 1000 µM ZnCl_2_ (Figure 2C), HGT-1 TAS2R43ko cells were used to investigate the functional role of TAS2Rs in the cellular uptake of zinc. The measured concentration of zinc in the TAS2R43ko cells (348.7 ± 46.6 fg/cell) was 40% higher than in the WT cells (226.7 ± 6.5 fg/cell) after exposure to 500 µM ZnCl_2_. At a concentration of 1000 µM, the intracellular zinc concentration in the TAS2R43ko (353.43 ± 56.3 fg/cell) cells exceeded the zinc levels in the WT cells by 31% (270.69 ± 28.4 fg/cell) (Figure 4B). Moreover, TAS2R39ko cells were measured with a cellular zinc concentration that was 15% higher than that of the WT cells (Figure S1C).

### 2.3. Impaired Cell Membrane Morphology of TAS2R43ko-Cells by Atomic Force Microscopy and TEER Analysis

**Figure 5:**
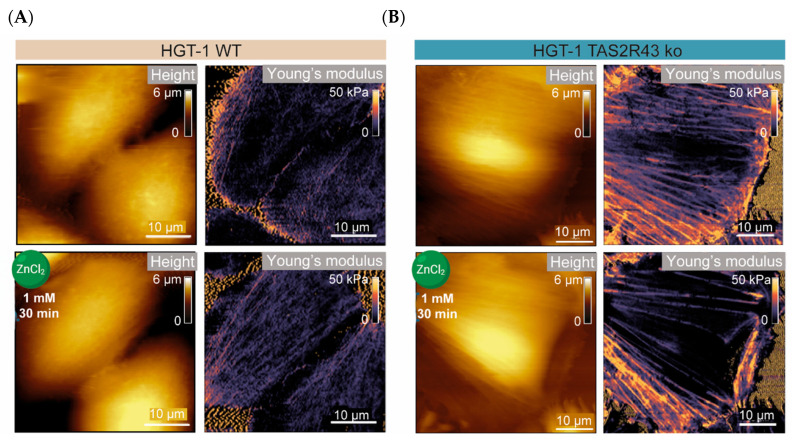
AFM results showing height and YM of HGT-1 WT and TAS2R43ko cells before and after the treatment with 1000 µM ZnCl2 for 30 min, with the height displayed in µm and the YM in kPa, in WT (**A**) and TAS2R43ko (**B**) cells (*n* = 4–6, t.r. = 3–5).

Focusing on the hypothesis that TAS2Rs are involved in the uptake of zinc, as indicated by the enhanced cellular zinc concentration observed in HGT-1 TS2R43ko cells (Figure 4B), the morphology of TAS2R43ko cells in comparison to the WT was investigated. Zinc is essential for membrane integrity [13] and can thereby affect cellular stiffness [35]. Likewise, changes in cellular stiffness may indicate alterations in membrane integrity that influence the function of membrane transport proteins [36], as well as modifications in cytoskeletal anchoring and cellular shape, e.g., changes in cell height. Measurement of the height and stiffness of HGT-1 WT and TAS2R43ko cells following zinc exposure was enabled by an AFM approach, which provided insights into zinc-induced morphological changes (Figure S4). A key parameter for assessing surface stiffness is the Young’s modulus (YM, in kPa) (example cells in Figure 5A,B), which is defined as the ratio between the stress (force per unit) and strain (proportional deformation) [37]. A higher YM indicates a stiffer cell.

**Figure 6:**
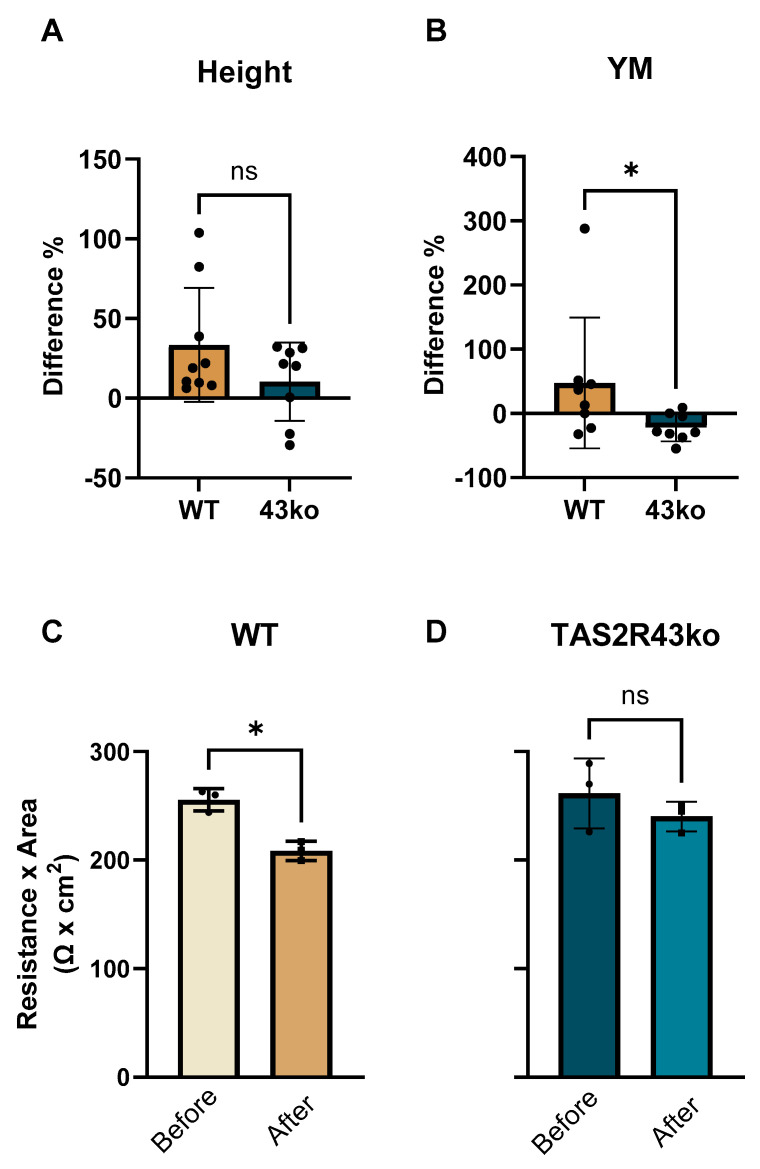
Comparison of the differences in the relative changes in height (**A**) and stiffness (**B**) of WT and TAS2R43ko cells upon treatment with 1000 µM ZnCl_2_, displayed in % (normed on the values before the treatment, each dot representing a measurement). Comparison of TEER as resistance × area in HGT-1 WT (**C**) and TAS2R43ko (**D**) cells upon treatment with 1000 µM ZnCl_2_ (*n* = 3–4, t.r. = 3). Statistics performed using Mann–Whitney test with significance displayed as * = *p* ≤ 0.05 and ns = not significant.

The HGT-1 WT cells reacted to the treatment by rising in height by 28%, from 2.9 ± 0.6 µm to 3.7 ± 0.8 µm (*p* < 0.05), without changing their volume (Figure S5A). Notably, the height of the TAS2R43ko cells did not change in response to the ZnCl_2_ treatment (*p* = 0.1964) (Figure S5B). In contrast, the YM of the WT cells remained unaltered by the treatment (before: 8.1 ± 3.9 kPa; after: 8.3 ± 2.9 kPa; *p* = 0.8876), but the TAS2R43ko cells responded with a reduction of 20% (from 14.4 ± 3.1 kPa to 11.6 ± 4.9 kPa; *p* < 0.05) (Figure S5C,D). The relative change in height between the WT cells compared and the TAS2R43ko cells did not reach a statistically significant difference (*p* = 0.48) despite its visible trend (Figure 6A). The difference in the YM is displayed in Figure 6B, exhibiting the reduced stiffness of TAS2R43ko cells.

TEER experiments complement AFM measurements by providing insights into cell layer integrity, barrier function, and mechanical properties. Together, these methods complete the picture of how zinc treatment affects cell morphology. By measuring the electrical resistance of the cell’s monolayer, TEER reveals the integrity of tight junctions and the cell’s overall permeability. For consistency, HGT-1 WT and TAS2R43ko cells were treated after reaching a confluent barrier on day 4 post-seeding, with the confluence being confirmed by Lucifer yellow staining. Treatment with ZnCl_2_ (0–1000 µM) produced similar effects for both the HGT-1 WT and TAS2R43ko cells across all concentrations (Table S3). However, a significant reduction in the TEER values, from 256 to 209 Ω × cm^2^ (decrease of 18%, *p* < 0.5), was observed only in the HGT-1 WT cells that were exposed to 1000 µM ZnCl_2_ (Figure 6C). This decrease in TEER values, combined with cellular softening, suggests that zinc exposure may weaken the barrier by disrupting the cell’s cytoskeleton or compromising its tight junction integrity. The TAS2R43ko cells exhibited a reduction in stiffness while maintaining their TEER (Figure 6D). ICP-MS quantitation showed less than <0.4% of zinc (Table S4) migrating from the apical to the basal compartment after the treatment (0–1000 µM ZnCl_2_), indicating that zinc uptake occurs predominantly within the cells rather than through passive diffusion across the monolayer.

### 2.4. Increased Protein Expression of Zinc Transporter ZIP14 in HGT-1 TAS2R43ko vs. WT-Cells

Given that the TAS2R43ko cells showed higher intracellular zinc levels compared to the WT cells, while maintaining cellular integrity, albeit with reduced stiffness, it was hypothesized that the response of ZTPs might be affected. ZTPs, including the ZnT and ZIP families, are critical for sustaining zinc homeostasis. To assess potential changes in ZTP expression, as well as alterations in calcium binding proteins in gastric parietal cells, a human genome-wide screening was performed using customized cDNA microarrays, with which we compared cells exposed to 1000 µM ZnCl_2_ with untreated control cells. This screening revealed responses of high magnitude for several genes, including ZIP2, ZIP14, ZnT1, ZnT5 A, ZIP11, ZIP3, GPR39, and the calcium binding proteins S100 A1 and S100 G (Table S5).

In a subsequent qPCR experiment, primers for the zinc importing transporters ZIP4, ZIP5, and ZIP14 and the cellular zinc exporters ZnT1 and ZnT5b were selected according to their reported occurrence in the gastro-intestinal tract and their membrane localization [16,38,39,40]. All tested ZTP genes were adequately expressed with dCt values ranging between 6 and 11 (Figure S6A). The baseline expression of these genes was similar in untreated HGT-1 WT and TAS2R43ko cells (Figure S6A,B); however, the FC differed between the cell types for ZnT1 and ZIP14 at 100 µM, and for ZIP5 at 500 µM ZnCl_2_ (Figure S6B).

**Figure 7:**
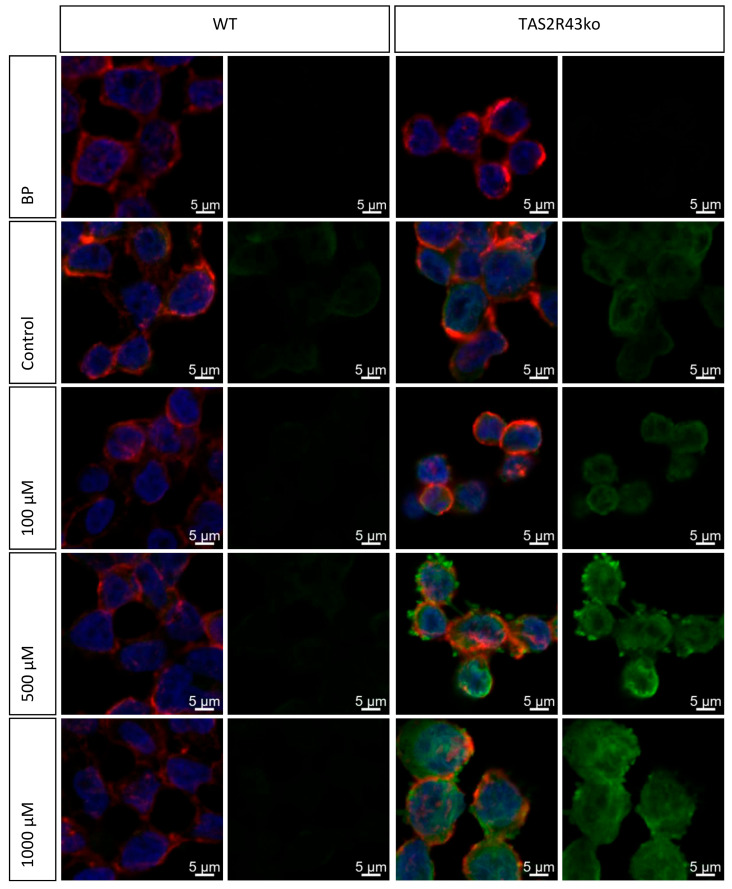
Immuno-cytochemistry staining (ICC) of ZIP14 in HGT-1 WT (**left**) and TAS2R43ko (**right**) cells as overlay and the channel of the Zn-transporter after the treatment with ZnCl_2_ (100, 500, and 1000 µM) for 30 min prior to the staining procedure. Fluorescence dyes: nucleus in blue (Hoechst), cell membrane in red (Alexa 633), Zn-transporter in green (Alexa 488) (*n* = 3–4, t.r. = 4–6).

To validate the impact of TAS2R43 on the protein levels of ZTPs, an ICC staining was conducted for the selected ZTPs, ZIP4, ZIP14, ZnT1, and ZnT5. The fluorescence intensities, quantitated as the corrected total cell fluorescence (CTCF), enabled a direct numerical comparison between the HGT-1 WT and TAS2R43ko cells (Figures S7 and S8A–D, Table S6). The antibody specificity was confirmed by a blocking peptide that reduced the fluorescence intensity (Figure 7).

**Figure 8:**
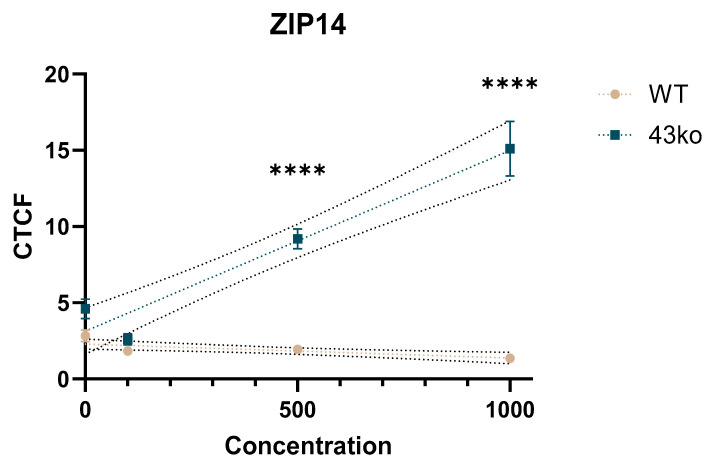
CTCF of ZIP14 on WT and TAS2R43ko cells after the treatment with ZnCl_2_ as mean ± SD with linear fit. Statistical difference between WT and TAS2R43ko cells tested by two-way ANOVA and displayed as **** = *p* ≤ 0.0001 (*n* = 3–4, t.r. = 4–6).

Notably, the TAS2R43ko cells exhibited significantly higher ZIP14 intensities compared to the WT cells, with mean FCs of 4.7 and 11.1 when exposed to 500 and 1000 µM of ZnCl_2_, respectively (Figure 8). These results suggest that the enhanced intracellular zinc concentration observed in the TAS2R43ko cells may be caused by the upregulation of ZIP14 at the protein level, potentially contributing to altered zinc homeostasis in these cells.

## 3. Discussion

We hypothesized that gastric TAS2Rs regulate both PSA, as a key mechanism of GAS, and zinc homeostasis. To test this hypothesis, we examined the role of bitter taste receptors in modulating PSA in response to zinc salts and the intracellular zinc levels in immortalized human parietal cells, thereby revealing broader physiological functions of TAS2Rs beyond oral bitter taste perception. Our findings demonstrate that TAS2R43, in particular, plays a critical role in modulating PSA and controlling intracellular zinc levels, which may contribute to cellular protection against metal-induced stress. To our knowledge, this is the first study to directly address a link between zinc and TAS2Rs.

The zinc concentrations used throughout the experiments ranged from 100 up to 1000 µM of ZnCl_2_, with the latter representing the consumption of highly dosed supplements (30 mg) on an empty stomach. The inhibitory effect of zinc on GAS has been reported previously for concentrations of 300 µM [28], yet the underlying mechanism remained unclear. Our proton secretion assays confirmed that zinc, applied either as ZnCl_2_ or ZnSO_4_, reduces PSA. The similar responses observed for both salts implicate the zinc cation as the active agent (Figure 1). Given that TAS2Rs are known to co-regulate acid secretion in parietal cells [2], we screened for TAS2R expression in HGT-1 cells, as gene expression has previously been linked to the receptor’s functionality [5,31]. RT-qPCR revealed the expression of most TAS2Rs in untreated gastric parietal cells, except for TAS2R60, 41, 7, 9, 38, and 1, which had Ct values over 38 and were not pursued further (Figure 2A) [2]. Among the six receptors responding to zinc (Figure 2C), TAS2R39 exhibited the most pronounced fold change, despite a comparably low expression (dCt = 19.17), while TAS2R43, being highly expressed (dCt = 14.59), was unique in showing a concentration-dependent correlation between its ddCt values and PSA (Figure 3A). Subsequent knockout experiments revealed that, while the HGT-1 TAS2R39ko cells maintained PSA levels similar to those of the WT cells (Figure S2A), the TAS2R43ko cells exhibited a higher PSA upon exposure to 1000 µM ZnCl_2_ (Figure 3B). This supports the assumption of a specific role for TAS2R43 in mediating zinc’s inhibitory effect on mechanisms of gastric acid secretion. These findings are consistent with previous reports identifying TAS2R43 to reduce prosecretory activity in parietal cells treated with bitter tasting caffeine [2].

To further explore potential mechanisms supporting our first hypothesis that TAS2Rs are involved in the zinc-mediated inhibition of PSA, we examined intracellular calcium mobilization using Cal-520. Ca^2+^ is a well-known second messenger that facilitates GAS via a TAS2R-dependent signaling pathway [41], meaning that its mobilization could provide clues about how TAS2Rs affect proton secretion. Additionally, Hershfinkel et al. (2001) demonstrated that zinc induces via a yet-unknown GPCR, sensing Zn^2+^, the release of Ca^2+^ from intracellular stores through IP_3_ in HEK293 cells [42]. The authors demonstrated that this release was specific to zinc and did not occur in response to other (heavy) metals. Furthermore, TAS2Rs have been shown to trigger Ca^2+^ release, via an IP_3_-mediated cascade, into the cytosol [43]. Moreover, there is evidence that zinc activates the transient receptor potential channel of the subfamily mucolipin (TRPML1), resulting in cellular Ca^2+^-efflux in HGT-1 cells [44], which agrees with our data. In our study, the Cal-520 assay revealed that the HGT-1 WT cells released less calcium compared to the TAS2R43ko cells, establishing the involvement of TAS2R43. Despite the potential limitation that the calcium-sensitive dye may interact with divalent zinc ions [45,46], the distinct differences between WT and TAS2R43ko cells remain.

**Figure 9:**
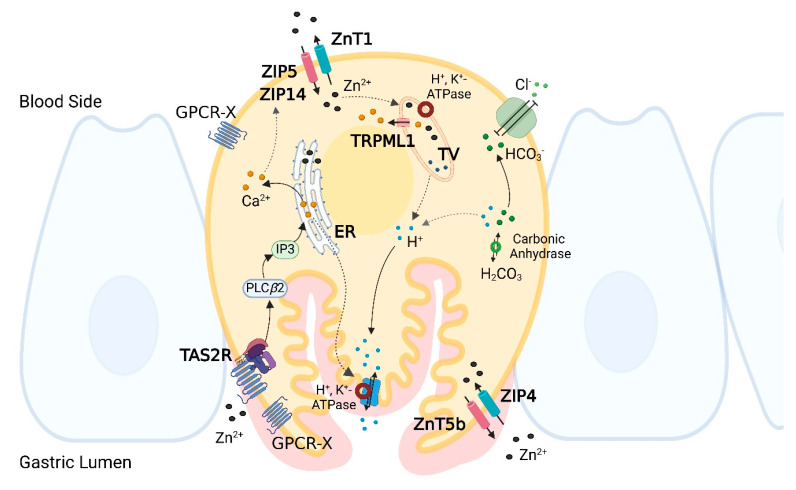
Signaling cascade via TAS2Rs in human parietal cell after exposure to zinc mediating cellular Zn^2+^ concentration and PSA. Arrows with solid lines are based on literature data; dashed arrows represent hypotheses derived from this study (TV = tubulovesicles; ER = endoplasmic reticulum). Created with BioRender.com.

The cDNA microarray screening further provided insights into potential mechanisms. On one hand, a decreased activity of H^+^, K^+^ ATPase would reduce proton exchange and secretion. On the other hand, a reduction in carbonic anhydrases could disturb the equilibrium of carbonic acid with H^+^ and HCO_3_^−^. Since HCO_3_^−^ acts as an exchange ion for Cl^−^ influx, such alterations could interfere with proton secretion [47]. Figure 9 presents a schematic of a possible TAS2R-mediated mechanism of PSA upon zinc exposure, incorporating the yet-unknown GPCR proposed by Hershfinkel et al. [48] as “GPCR-X”.

Liu et al. (2011) discovered that intracellular Ca^2+^ modulates the basolateral uptake of zinc ions, suggesting that zinc accumulation may depend on intracellular Ca^2+^ by facilitating the exchange of intracellular Ca^2+^ for extracellular Zn^2+^ [49]. In addition, Naik et al. [22] described a physiological link between luminal acid secretion and the Zn^2+^ content in tubulovesicles, serving as secretory compartments. With Duarte et al. [8] demonstrating that TAS2R14 regulates the transport of resveratrol in human choroid plexus epithelial cells through blood–cerebrospinal fluid barriers, our second hypothesis, addressing the potential role of TAS2Rs in the uptake of small molecules, comes into focus. Although zinc uptake in parietal cells has not been extensively investigated, our study shows that the intracellular zinc concentration in HGT-1 cells increases dose-dependently with exposure to zinc salts (Figure 4A). Importantly, HGT-1 TAS2R43ko cells exhibited higher intracellular zinc concentrations than WT cells (Figure 4B and Figure S1C), suggesting that TAS2R43 might normally limit zinc uptake, thereby protecting cells against excessive intracellular zinc accumulation. Yet, the mechanism by which zinc enters the cell in the absence of TAS2R43 remains to be elucidated.

A first approach to address this question involved assessing the cells’ morphology via AFM, providing first insights into membrane architecture. As shown in Figure 5, the overall morphology of HGT-1 WT cells remained largely unchanged upon zinc exposure, except for an increase in height (Figure S5A). In contrast, the TAS2R43ko cells exhibited a decreased YM, indicating cell softening, while their height remained unchanged (Figure S5). Given that the actin cytoskeleton is a major determinant of cellular mechanical properties, and that the weakening of actin filaments leads to decreased stiffness [50], these findings suggest that TAS2R43ko cells may lack the tension required for growth in height. Notably, it is not yet known whether zinc preferentially binds to the cell surface of intact WT cells rather than accumulating intracellularly. Moreover, alterations in the cytoskeleton may impact the membrane environment of ion channels and transporters, thereby affecting the cell’s permeability. Although the literature on the application of AFM to cell culture is limited, our complementary TEER measurements indicated that, while the WT cells experienced an 18% reduction in TEER at 1000 µM ZnCl_2_, the TAS2R43ko cells maintained their TEER. The reduced TEER values without changes in stiffness indicates a weakening of tight junction integrity in WT cells, which potentially increases their permeability without markedly altering their mechanical properties. In contrast, TAS2R43ko cells exhibited a reduction in stiffness while maintaining TEER (Figure 6D), implying that zinc in these cells primarily affects cytoskeletal organization rather than directly disrupting tight junctions. This difference might be linked to TAS2R43-mediated signaling pathways influencing cytoskeletal remodeling or cell–cell adhesion dynamics. Moreover, despite the observed differences in the TEER and mechanical properties between the HGT-1 WT and TAS2R43ko cells, the minimal zinc migration (<0.4%) suggests that the overall barrier function remains largely intact, even at high zinc concentrations.

Finally, to elucidate the molecular basis for the altered zinc homeostasis observed in TAS2R43ko cells, we conducted a genome-wide cDNA microarray screening followed by targeted qPCR and immunocytochemistry (ICC). The screening identified several zinc transporter proteins (ZTPs), including ZIP2, ZIP14, ZnT1, ZnT5 A, ZIP11, ZIP3, and GPR39, as well as calcium-binding proteins, H^+^, K^+^-ATPases, and carbonic anhydrases, that responded robustly to 1000 µM ZnCl_2_ treatment (Figure S6). Although the literature on the calcium binding protein S100 G is limited, its function as a calcium buffer is recognized [51], while S100 A1 has been reported as a dominant negative regulator for the binding of Zn^2+^ to S100B [52]. As mentioned above, Hershfinkel et al. [42] discussed the zinc-dependent release of calcium in HEK293 cells, so that alluding the calcium binding proteins appears reasonable. The exact transport mechanisms of zinc in cells are still not fully elucidated, but the involvement of ion transporters of the ZIP and ZnT1 family is commonly agreed on [15,28,42]. To date, no association between TAS2Rs and ZTPs has been described in the literature.

In targeted qPCR experiments, the main zinc transporter genes found in gastric cell lines, ZIP4, ZIP14, ZIP5, ZnT1, and ZnT5b, all exhibited altered expression upon ZnCl_2_ exposure. ICC analyses of ZIP4, ZIP14, ZnT1, and ZnT5b revealed that the ZIP14 protein expression was markedly upregulated in the TAS2R43ko cells compared to the WT cells (Figure 7 and Figure 8), while the other transporters showed low or nonspecific staining (Table S6). The increased ZIP14 expression in TAS2R43ko cells likely contributes to their higher intracellular zinc levels, suggesting an association between TAS2R43 activity and ZIP14-mediated zinc import.

Considering that TAS2Rs activation leads to Ca^2+^ release, which in turn may facilitate zinc uptake via ZIP14, Steimle et al. [53] proposed that elevated intracellular Ca^2+^ enhances the uptake of divalent metal ions by modulating the localization of zinc transporters such as ZIP8 or ZIP14. In our study, the increased Ca^2+^ mobilization observed in TAS2R43ko cells may thus account for their higher zinc accumulation due to enhanced ZIP14 activity. Collectively, these results provide initial insights into mechanisms by which TAS2Rs might protect parietal cells from excessive intracellular zinc accumulation.

It should be noted, however, that HGT-1 cells represent a transformed tumor-derived in vitro model and may not fully replicate the in vivo physiology of native human gastric parietal cells. Nevertheless, the physiological relevance of our findings is supported by in vivo studies demonstrating that GAS correlates with PSA, thereby validating our in vitro observations [2]. Future studies are needed to further elucidate these mechanisms in humans and to determine whether they are specific to zinc or extend to other dietary metals.

## 4. Material & Methods

### 4.1. Chemicals

Fetal bovine serum (FBS), penicillin-streptomycin (PS), and trypsin/ethylendiaminetetraacetic acid were obtained from PAN Biotech GmbH (Aidenbach, Germany). 1,5-Carboxy-seminaphtorhodaflour acetoxymethylester (SNARF-1AM), Gibco Dulbecco’s modified Eagle’s medium Glutamax (DMEM), Nigericin, and Pluronic F127 were acquired from Thermo Fisher Scientific (Darmstadt, Germany). Dimethylsulfoxide (DMSO), KCl (99.999%), NaCl (99.99%), and thiazolylblue were ordered from Carl Roth (Karlsruhe, Germany). 3-(4,5-dimethylthiazol-2-yl)-2,5-diphenyltetrazolium bromide (MTT), 4-(2-hydroxyethyl)-1-piperazineethanesulfonic acid (HEPES) (≥99.5%), D-glucose (≥99.5%), CaCl_2_ (≥99.995%), and KOH were obtained from Merck KGaA (Darmstadt, Germany). ZnCl_2_ (≥99.91%) was received from VWR (Leuven, Belgium) and ZnSO_4_ (≥99.95%) from Sigma-Aldrich (St Louis, MO, USA). Phosphate-buffered saline was bought from Biozym Scientific GmbH (Hessisch Oldendorf, Germany). Propidium iodide (PI) was obtained from Miltenyi Biotec (Bergisch-Gladbach, Germany), calibration- and cleaning solutions for the ICP-MS Nexion 5000 were purchased from Perkin Elmer (Rodgau, Germany). Material for qPCR experiments, including customized plates for TAS2Rs, were obtained from BioRad (Feldkirchen, Germany), while the primers for zinc transporter were synthesized by Eurofins (Ebersberg, Germany). Antibodies for immunocytochemistry (ICC) were purchased together with the corresponding blocking peptides from Alomone Labs (Jerusalem, Israel). The dyes Hoechst-33342, Streptavidin Alexa Fluor^TM^ 633 conjugate, and Alexa Fluor 488 anti-Rabbit IgG were received from Fisher Scientific GmbH (Schwerte, Germany). Concanavalin A biotin conjugate came from Sigma-Aldrich (Munich, Germany). The fluorescent dye Cal-520 was obtained from Biomol (Hamburg, Germany). The C1 buffer used contained 130 mM NaCl, 10 mM HEPES pH 7.4, 5 mM KCl, 2 mM CaCl_2_, 0.18% glucose.

### 4.2. Cell Culture

The human gastric tumor cell line (HGT-1) used in cell culture experiments was purchased from Merck (Merck KGaA, Darmstadt, Germany). The cells were cultured under standard conditions in DMEM, supplemented with 10% FBS and 1% PS at 37 °C and 5% CO_2_. Cells of the wild type (WT) were used in passages between 16 and 32.

Stable CRISPR-knockout (ko) cells of TAS2R43 were used according to the previously published protocol [2]. Homozygous TAS2R39 ko was generated (Figure S2) by applying a general CRISPR-Cas9 protocol used for generating a homozygous TAS2R43, as described previously [6], with the following modifications: For transfection, a total of 15,000 HGT-1 cells per well were seeded on a 96-well plate and allowed to settle for 24 h. Transfection was conducted using Invitrogen TrueGuide Synthetic sgRNA targeting TAS2R39 gene U*U*C*CACAAGUGGCAGGAUCC+ modified scaffold (5′−3′) or, as a control, a nontargeting TrueGuide sgRNA negative control, according to the Invitrogen TrueCut Cas9 Protein v2 protocol, for 48 h. Lysis of the cells and DNA extraction were performed according to the manufacturer’s protocol. The cleavage efficiency was analyzed using a Genomic Cleavage detection kit (Thermo Fisher Scientific). The following primers were used for genomic cleavage detection: forward primer CCTTCCAACATTTGCATCTC (5′−3′) and reverse primer GTAGAAGAAACTGAGCCA (5′−3′). The following temperature protocol was used: 95 °C/10 min, 40 cycles of 95 °C/30 s, 55 °C/30 s, 72 °C/30 s, and finally 72 °C for 7 min. Before the single-cell cloning of transfected cells, the transfected cells were cultivated in a T25 cell culture flask to obtain a higher cell count. Single cells were cloned in 96-well plates and, after reaching confluence, transferred to a 24-well plate and later to a 6-well plate before each clone was frozen in cryotubes. DNA was extracted with a PureLink Genomic DNA Mini Kit (Life Technologies, Darmstadt, Germany) according to the manufacturer’s protocol, and clones were amplified with AmpliTaqGold 360 Mastermix (Thermo Fisher Scientific). Subsequent PCR was carried out as described earlier for the genomic cleavage detection followed by Sanger sequencing to identify deletion on an mRNA level conducted by Eurofins Genomics (Vienna).

The two zinc salts ZnCl_2_ and ZnSO_4_ were chosen to verify a cellular effect being due to zinc, and independent of the counter ion. Both zinc salts were applied in physiologically relevant concentrations, which mirror those that would be reached after ingesting zinc supplements with 30 mg on an empty stomach. The cells’ incubation for 30 min was based on the retention time of 30 min in the stomach [54]. Generally, the cells were treated with ZnCl_2_ or ZnSO_4_ dissolved in FBS- and PS-free DMEM for 30 min under standard conditions of 37 °C and 5% CO_2_, and then harvested with trypsin/EDTA if not declared otherwise.

### 4.3. Cell Viability Assay

The metabolic activity of HGT-1 cells was tested for every treatment to exclude cytotoxic effects of the test compounds in their relevant concentrations. For this purpose, cells were seeded for 20–24 h in transparent 96-well plates at a density of 50,000 cells/well, and were then stained under standard conditions for 10 min with 100 µL MTT dye (0.83 mg/mL in DMEM without FBS or PS) after 30 min exposure to solutions of 100–1000 µM ZnSO_4_ or ZnCl_2_. The emerging formazan was diluted in DMSO and analyzed for its absorbance with an Infinite M200 plate reader (Tecan, Switzerland) at a wavelength of 570 nm and a reference of 650 nm. Cell viability was calculated based on the absorbance in comparison to untreated cells (100%).

### 4.4. Proton Secretory Activity (PSA)

The measurement of PSA enables the identification of bitter and potentially toxic compounds by the modulation of PSA as key mechanism of GAS [2], thereby conducting a defense function (reviewed by Behrens et al. [55]). HGT-1 WT cells, CRISPR-Cas9 TAS2R39ko cells, and TAS2R43ko cells were seeded in black 96-well plates at a density of 50,000 cells/well 20–24 h prior to the experiment. Afterwards, the cells were washed with C1 buffer, incubated with solutions of ZnCl_2_ or ZnSO_4_ [54], as well as MgSO_4_, KCl, Na_2_SO_4_, or NaCl (100–1000 µM [56]), and washed again before 30 min incubation with 3 µM pH-sensitive SNARF-1AM dye in C1 buffer. The dye was removed, after which the cells were washed and covered with C1 buffer. Potassium buffer (20 mM NaCl, 110 mM KCl, 1 mM CaCl_2_, 1 mM MgSO_4_, 18 mM D-glucose, and 20 mM HEPES) containing 2 µM nigericin was added for calibration (pH range 7.0–8.0). As shown in previous research [57], histamine (1 mM in C1 buffer) was used as positive control as it does not target TAS2Rs. A Flexstation3 (Molecular Devices, San Jose, CA, USA) was used to monitor the fluorescence emission at the wavelengths of 580 and 640 nm after excitation of the dye at a wavelength of 488 nm. By calculating the 580/640 nm absorption ratio relative to that of untreated control cells subtracted from 1, which was used as reference value of control cells, we quantitatively described the changes in PSA.

### 4.5. TAS2R Gene Expression

Quantitation of TAS2R and zinc transporter proteins (ZTPs) mRNA expression levels in HGT-1 cells was performed by real-time-qPCR (RT-qPCR) according to Richter et al. [5]. Briefly, 800,000 cells were seeded 20–24 h prior to the experiment. The cells were incubated with ZnCl_2_ solution (100–1000 µM), harvested using a lysis buffer enriched with 2-Mercapto-Ethanol, and subjected to RNA isolation using the peqGOLD RNA Kit (VWR Peqlab, Radnor, PA, USA). The mRNA concentration was determined using a NanoDrop One (A260/280) (Thermo Fisher Scientific, Waltham, MA, USA). gDNA was removed and cDNA synthesis was completed with an iScript gDNA Clear cDNA Synthesis Kit (Biorad, Feldkirchen, Germany). RT-qPCR was carried out by amplifying 50 ng of cDNA with SsoAdvanced Universal SYBR Green Supermix (Biorad, Feldkirchen, Germany) on 25 TAS2Rs as described by Liszt et al. [2] (Table S7). Additional RT-qPCR-experiments were performed with primers for the following ZTPs: ZnT1, ZnT5b, ZIP4, ZIP5, and ZIP14. GAPDH and PPIA were used as reference genes. Data were calculated with the ddCt method as fold change (FC).

### 4.6. Calcium Assay Using Cal-520

Ca^2+^ plays a major role in the intracellular signaling cascades that occur upon activation of TAS2Rs so that measuring its mobilization gives evidence on the predominant pathway of proton secretion [58]. Intracellular calcium mobilization was characterized by means of the calcium-sensitive fluorescent dye Cal-520 [43]. HGT-1 WT and TAS2R43ko cells were seeded with a density of 50,000 cells/well 20–24 h prior to the experiment. After washing with C1 buffer, the cells were stained for 120 min with 1.0 µM Cal-520 (in C1 buffer with 0.004% Pluronic F-127 and 0.02% DMSO) under standard conditions. The dye was aspirated, the cells washed, covered with C1 buffer, and set for measurement in 1 s interval using a Flexstation3 at 37 °C (excitation: 493 nm, emission: 515 nm). ZnCl_2_ was added at concentrations of 100, 500 and 1000 µM 60 s after the measurement started. Ionomycin was used as positive control at a concentration of 1 µM. Fluorescence intensity was normalized to the first 60 s before the test compounds were added. Additionally, the dye was tested for its reaction with the zinc solutions in a blind setting without cells [44].

### 4.7. Intracellular Zinc Concentration

To quantify the cellular zinc concentration, cells were measured in a bulk setup with inductively coupled mass spectrometry (ICP-MS), based on the protocol of Meyer et al. [59] with minor modifications for analyzing zinc in HGT-1 cells. Cells were seeded at a density of 1 × 10^6^ in T25 cell culture flasks 20–24 h prior the experiment. They were washed with C1 buffer, incubated with ZnCl_2_ or ZnSO_4_ (100, 250, 500, or 1000 µM), harvested, and washed again three times with C1 buffer. Using a MACSQuant Flow Cytometer (Miltenyi Biotec, Bergisch-Gladbach, Germany) operating at 488 nm, the cell count was recorded. Propidium iodide functioned as fluorescent dye to track the cells’ viability during the experiment. For microwave digestion (Anton Paar, Courtaboeuf, France), 1 mL of cell suspension with 4 mL of ddH_2_O and 1 mL of HNO_3_ (69%) was pipetted into a sealed vessel and digested as follows: 20 min ramp to 190 °C, hold for 20 min, 20 min cool down. The digests were transferred quantitatively into 25 mL volumetric flasks and filled up to this volume with ddH_2_O. Finally, the content of zinc was quantitated in the digests as well as in the supernatants by measuring ^66^Zn with a Nexion 5000 ICP-MS in MS/MS mode and ammonia DRC profile with following instrument parameters set after a daily smart tune with NEXion setup solution: ICP-RF power: 1500 W; plasma gas flow: 16 L/min; ammonia flow rate: 0.3 L/min; auxiliary gas flow rate: 1.2 L/min; nebulizer gas flow: 0.86–0.88 L/min; RPq = 0.6; dwell time per amu: 50 ms; IGM: focusing. A solution of 10 µg/L scandium acted as internal standard, automatically added to the sample by the instrument. The amount of metal per cell was calculated with respect to the determined cell counts. Data were processed using Syngistix v.3.3 (Perkin Elmer, Waltham, MA, USA).

### 4.8. cDNA Microarrays

For a gene expression analysis screening [60], HGT-1 cells were seeded by 800,000 cells in T25 cell culture flasks 20–24 h prior to the experiment. After exposing the cells to ZnCl_2_ (100 and 1000 µM) the cells were washed twice with 500 µL C1 buffer and harvested using 500 µL lysis buffer enriched with 10 µL 2-mercapto-ethanol. Following the manufacturer’s protocol, RNA was isolated and cleaned using the peqGOLD RNA Kit (VWR Peqlab, PA, USA). The RNA concentration was determined by a NanoDrop One (A260/280) (Thermo Fischer Scientific, USA) before gDNA was removed using EZDNase (Invitrogen, Thermo Fisher Scientific). First strand synthesis and reverse transcription of the RNA was performed with Superscript IV (Invitrogen) and nonamer primers (Tebu Bio). After purification (QIAquick purification columns, Qiagen, Hilden, Germany), the samples were dried and stored at −80 °C for a maximum of 2 weeks until further use. For analysis, cDNA was resuspended in 12 µL hybridization mix containing 6 µL of MES buffer, 0.7 µL acetylated BSA (10 mg/mL), 0.13 µL herring sperm DNA (10 µg/mL), 0.45 µL QC25-Cy-3 (100 nM), 0.45 µL EcoBioA1 (100 nM), 0.45 µL EcoBioD2 (100 nM), and 3.85 µL DNase free H_2_O. Subsequently, the samples were hybridized onto human gene expression microarrays for 21 h at 42 °C [60,61,62]. The slides were washed for 2 min in non-stringent wash buffer (SSPE; 0.9 M NaCl, 0.06 M phosphate, 6 mM EDTA, and 0.01% Tween20), for 1 min in stringent wash buffer (100 mM MES, 0.1 M NaCl, and 0.01% Tween20), and for 10 s in the final wash buffer (0.1× saline-sodium citrate buffer). The slides were spin-dried using a microarray centrifuge and, finally, scanned at 532 nm at 2.5 µM pixel-size resolution using an Axon GenePixPro microarray scanner (Molecular Devices).

### 4.9. Cell Membrane Height and Stiffness by Atomic Force Microscopy (AFM) as Well as Membrane Permeability by Trans-Epithelial Electrical Resistance (TEER)

A combination of an inverted epifluorescence microscope (Zeiss Axio Observer 7, 10× objective, NA = 0.95) and an AFM (NanoWizard V BioScience, JPK SPM Desktop v8.0.145, Bruker, Bremen, Germany) was used to obtain HGT-1 WT and TAS2R43ko cell images before and after treatment with ZnCl_2_. All experiments were performed under cell-culture conditions by operating the AFM at 37 °C (Temperature Controller, Bruker) using a special cell culture chamber perfused with synthetic air with CO_2_ (5%) at 95% relative humidity using a CO_2_ controller (CO_2_ –Controller 2000, Pecon).

PFQNM-LC probes (Bruker) with tip lengths of 17 μm, tip radii of 65 nm, and opening angles of 15° were used to record images of cell surfaces (20–50 μm^2^) and to probe their nanomechanical architecture (stiffness). The pre-calibrated spring constant was used to determine the deflection sensitivity using the thermal noise method [63] before each experiment. In PeakForce QNM mode, FD-based multiparametric maps were acquired using a force setpoint of 700 pN, with a cantilever oscillation frequency of 0.25–1 kHz (depending on desired resolution) and a peak-to-peak amplitude of 1.2 μm. The sample was scanned using a line rate of 0.22 and 128 or 256 pixels per line (256 lines). The measurements were performed as follows: HGT-1 WT and TAS2R43ko cells were cultured on 40 mm diameter petri dishes (TPP, Trasadingen, Switzerland) for 1 or 2 days before the experiment to ensure formation of a sufficient confluent monolayer. Guided by optical microscopy, at least 3 cells were scanned, then treated with 1000 μM ZnCl_2_, which was followed by a washing step, and then cells were scanned again.

AFM images were analyzed using the JPK data processing software (v8.0.145, Bruker) and Gwyddion (v2.62). For the topography images, a second-order plane fit was performed to obtain deconvoluted height images without further processing. For the nanomechanical probing data, the baseline of the retraction curve was corrected using a 2nd degree polynomial fit on the off-contact area to correct for the tilt sometimes present in raw FD curves [64,65].

To investigate the membrane integrity before and after the treatment with zinc, a TEER analysis was performed according to the protocol of Duarte et al. [8]. HGT-1 WT and TAS2R43ko cells were seeded at a density of 100,000 cells/well onto Transwell inserts (6.5 mm diameter, 0.4 µm pore size for 24-well plate) with a polyester membrane (Costar, Cornin Inc., New York, USA). Daily monitoring of the TEER with an EVOM Manual EVM-MT-03-01 (World Precision Instruments, Sarosata, FL, USA) was conducted to evaluate the integrity of the cell monolayer. A plateau was reached on day 4, on which the cells were exposed to ZnCl_2_ (100, 500, and 1000 µM). Afterwards, the TEER was measured again, followed by a permeability assay using Lucifer yellow in HBSS (PAN GmbH, Biotech, Aidenbach, Germany) to confirm the integrity of the cell monolayer. To test if zinc diffused from apical to basal compartments, the zinc concentrations in the supernatants of both compartments were quantitated by means of ICP-MS (2.8).

### 4.10. Immunocytochemistry Staining

Fluorescence labelling [43] of ZTPs in HGT-1 cells was performed on 10-well microscope slides precoated with 10 µg/mL poly-D-lysine in ddH_2_O. Briefly, HGT-1 WT cells were seeded at a density of 100,000 cells/well and HGT-1 TAS2R43ko cells at a density of 150,000 cells/well 20–24 h prior the experiment. After the treatment with ZnCl_2_, (100–1000 µM), the cells were stained using biotin-conjugated concanavalin A in PBS for 60 min, fixated by incubation with 4% formaldehyde for 10 min, and treated with 0.5% Triton X-100 A 45 min incubation with block solution (PBS/5% horse serum/0.3% Triton X-100) to reduce the level of unspecific binding. The cells were treated with primary antibodies (1:100: 80 µg/mL) anti-ZnT-1 (SLC30A1; Cat: AZT-011) or anti-ZIP14 (SLC39A14; Cat: AZT-024) and their corresponding blocking peptides (1:100: 160 µg/mL) for 60 min in blocking solution. The cell membrane was labelled with Strep-Alexa 633, the zinc transporter with anti-IgG rabbit, and Strep-Alexa 488 and the nucleus was dyed with Hoechst. Embedded in Fluoromount-G^TM^, the slides were stored at 4 °C until examination on a spectral confocal microscope on the next day. Using a LSM 780 (Zeiss, Oberkochen, Germany) microscope with a 40×/1.2 Imm Korr DIC 27 objective lens, images of the LSM T-PMT detector were acquired. Pictures were created with the ZEN 2.3 SP1 black program, and the intensities of the staining were analyzed with ImageJ (v1.54i) to determine corrected total cell fluorescence (CTCF).

### 4.11. Statistical Analysis

Each experiment was conducted with at least three biological replicates (b.r.) and 3–6 technical replicates (t.r.). For statistical analysis, outliers were excluded after Nalimov outlier analysis. Additionally, all data have been verified for normality distribution using Shapiro–Wilk test and/or Kolmogorov–Smirnov test. Statistical significance was considered for *p*-values smaller than 0.05, according to one-way ANOVA and Holm–Sídák post hoc test or Student’s *t*-test using GraphPad Prism (v10.4.2). Correlations were calculated after testing the distribution according to Pearson correlation coefficients. Data from cDNA microarrays was analyzed via R using custom annotation and design package as described by Danzer et al. [66]. Data were displayed as mean ± SD. *p*-values were indicated with asterisks according to the following scheme: a* = *p* ≤ 0.05, ** = *p* ≤ 0.01, *** = *p* ≤ 0.001, **** = *p* ≤ 0.0001.

## 5. Conclusions

Our work demonstrated that parietal cells assimilate Zn^2+^, while its cellular uptake involves numerous mechanisms through which TAS2Rs have been demonstrated to mediate cellular zinc homeostasis. Therefore, TAS2R43 might be attributed to a defensive function against excessive zinc accumulation in immortalized, human parietal cells. Whether this mechanism plays a key role for humans exposed to high, potentially toxic zinc concentrations and whether it applies to other essential divalent trace elements in foods has to be elucidated in future studies.

## Data Availability

Data will be made available by the corresponding author on reasonable request.

## Supplementary Materials

The following supporting information can be downloaded at: https://www.mdpi.com/article/10.3390/ijms26136017/s1.
