# Supplementary material for: Bitter Taste Receptor TAS2R43 Co-Regulates Mechanisms of Gastric Acid Secretion and Zinc Homeostasis
Source: Int J Mol Sci. 2025 Jun 23;26(13):6017. doi: 10.3390/ijms26136017 (PMC12249961; doi:10.3390/ijms26136017)
Supplement: Supplementary file 1 [file ijms-26-06017-s001.zip › ijms-3602778-supplementary.pdf]

Supplemental Material to

Bitter Taste Receptor TAS2R43 Co-regulates Mechanisms of Gastric Acid Secretion and Zinc Homeostasis

Noreen Orth[1,2], Philip Pirkwieser[2], Julia Benthin[1,2], Melanie Koehler[2,3], Sonja Sterneder[2,4,5], Etkin Parlar[6], Erika Schaudy[6], Jory Lietard[6], Timm Michel[1,2], Valerie Boger[2], Andreas Dunkel[2], Mark M. Somoza[2,6,7], Veronika Somoza[2,4,8]

[1] *Technical University of Munich, Graduate School of Life Sciences, Freising, Germany*

[2] *Leibniz-Institute for Food Systems Biology at the Technical University of Munich, Freising, Germany*

[3] *TUM Junior Fellow at the Chair of Nutritional Systems Biology, Technical University of Munich, Freising, Germany*

[4] *Department of Physiological Chemistry, Faculty of Chemistry, University of Vienna*

[5] *University of Vienna, Vienna Doctoral School in Chemistry (DoSChem), Währinger Str. 42, 1090 Vienna, Austria*

[6] *Department of Inorganic Chemistry, Faculty of Chemistry, University of Vienna, Vienna, Austria*

[7] *Chair of Food Chemistry and Molecular Sensory Science, School of Life Sciences, Technical University of Munich, Freising, Germany*

[8] *Chair of Nutritional Systems Biology, School of Life Sciences Weihenstephan, Technical University of Munich, Freising, Germany*

**Correspondence to Veronika Somoza: telephone +49-8161-71-2700,*

E-mail: v.somoza.leibniz-lsb@tum.de

Table S1: Sequences of TAS2Rs, zinc transporter Proteins and housekeepers for qPCR (Liszt et al., 2017; Liu et al., 2020; Zhu et al., 2021; Valentine et al., 2007; Jackson et al., 2007; Thornton et al., 2011).

Gene	Direction	5´to 3´ Sequence
TAS2R1	F	AAATGGCTCCGCTGGATCTC
	R	GTGGCAAGCCAAAGTTCCAA
TAS2R3	F	GGGACTCACCGAGGGGGTGT
	R	CCTCAAGAGTGCCAGGGTGGTG
TAS2R4	F	GCAGTGTCTGGTTTGTGACC
	R	GCGTGATGTACAGGCAAGTG
TAS2R5	F	ACACTCATGGCAGCCTATCC
	R	CGAGCACACACTGTCTTCCA
TAS2R7	F	GCAGGTGTGGATGTCAAACTC
	R	TCTTGACCCAGTCCATGCAG
TAS2R8	F	ATGTGGATTACCACCTGCCT
	R	GGAAATGGCAAAGCATCCCAG
TAS2R9	F	GCAGATTCGACTGCATGCTAC
	R	TGCCTTTATGGCCCTCATGT
TAS2R10	F	GCTACGTGTAGTGGAAGGCA
	R	TCCATTCCCCAAAACCCCAA
TAS2R13	F	GAAAGTGCCCTGCCGAGTAT
	R	CCAGATCAGCCCAATTCTGGA
TAS2R14	F	CCAGGTGATGGGAATGGCTTA
	R	AGGGCTCCCCATCTTTGAAC
TAS2R16	F	ATGGCATCACTGACCAAGCA
	R	TTTCAACGTAGGGCTGCTCA
TAS2R19	F	TCTTAGGACACAGCAGAGCA
	R	AGCGTGTCATCTGCCACAAAA
TAS2R20	F	ATTTGGGGGAACAAGACGCT
	R	ACTACGGAAAAACTTGTGGGAA
TAS2R30	F	GGCTGGAAAAGCAACCTGTC
	R	ACACAATGCCCCTCTTGTGA
TAS2R31	F	TTGAGGAGTGCAGTGTACCTTTC
	R	ACGGCACATAACAAGAGGAAAA
TAS2R38	F	CCCAGCCTGGAGGCCCACATT
	R	TCACAGCTCTCCTCAACTTGGCA
TAS2R39	F	TTCTGTGGCTGTCCGTGTTTA
	R	GGGTGGCTGTCAGGATGAAC
TAS2R40	F	CGGTGAACACAGATGCCACAGATA
	R	GTGTTTTGCCCCTGGCCCACT
TAS2R41	F	GCAGCGAATGGCTTCATTGT
	R	TGGCTGAGTTCAGGAAGTGC
TAS2R42	F	TCCTCACCTGCTTGGCTATC
	R	GGCAAGCCAGGTTGTCAAGT
TAS2R43	F	ATATCTGGGCAGTGATCAACC
	R	CCCAACAACATCACCAGAATGAC
TAS2R45	F	AGTACCCTTTACTGTAACCC
	R	AGTAAATGGCACGTAACAAG
TAS2R46	F	ACATGACTTGGAAGATCAAACTGAG
	R	AGCTTTTATGTGGACCTTCATGC
TAS2R50	F	CGCAAGATCTCAGCACCAAGGTC
	R	GCCTTGCTAACCATGACAACCGGG
TAS2R60	F	GGTGTTCAGTGCTGCAGGTA
	R	CACCTTGAGGAACGACGACT
ZnT1	F	CAATACCAGCAACTCCAACGG
	R	GCAAGGACCAGCCTCATAAAC
ZnT5B	F	GCGGGTGGAGGCATGAATGCTA
	R	TTTGGTTGTCTGTTTTACTTC
ZIP4	F	ATGTCAGGAGCGGGTCTTGC
	R	GCTGCTGTGCTGCTGGAAC
ZIP5	F	CTCATGCTTGCCATAACC
	R	AATCCTATTGCTCCTACTGG
ZIP11	F	TCTCCTAAGCATTTTGGTGGCCTA
	R	TCTCTTCTTTCCACAGGGCTCACT
ZIP14	F	ACTTAGGGCATCGCAGATGTTT
	R	GCAGTAACCTAGACGGGTCATTTAA
GAPDH	F	AGGTCGGAGTCAACGGATTTG
	R	GGGGTCATTGATGGCAACAATA
PPIA	F	CCACCAGATCATTCCTTCTGTAGC
	R	CTGCAATCCAGCTAGGCATGG

Table S2: ddCt of TAS2Rs tested for Pearson correlation against cellular zinc concentration and proton secretory activity.

TAS2R	Cellular zinc concentration vs. ddCt		Proton secretory activity vs. ddCt	
	r	*p*	*r*	*p*
14	0.9101	0.0899	0.8976	0.1024
19	0.9643	0.0357	0.9756	0.0244
30	0.8712	0.1288	0.8727	0.1273
39	0.8931	0.1069	0.8888	0.1112
45	0.9203	0.0797	0.9236	0.0764

Table S3: Proton secretory activity of HGT-1 TAS2R39ko cells upon treatment with $ZnCl_2$.

$ZnCl_2$ [µM]	0	100	500	1000
PSA TAS2R39ko cells	-0.02 ± 0.27	0.24 ± 0.31	0.49 ± 0.30	0.60 ± 0.41

Table S4: TEER values in Ω × cm^2 for HGT-1 WT and TAS2R43ko before and after the treatment with $ZnCl_2$ (0, 100, 500, and 1000 µM) for 30 min. Data shown as mean ± SD with n = 3-4.

		**0**	**100**	**500**	**1000**
**WT**	Before	250.33 ± 10.50	230.33 ± 25.66	249.67 ± 11.01	255.67 ± 10.21
	After	190.53 ± 14.76	185.27 ± 19.13	204.67 ± 2.89	208.76 ± 8.76
**TAS2R43ko**	Before	262.23 ± 23.46	265.00 ± 14.11	256.67 ± 19.86	261.67 ± 32.32
	After	199.27 ± 19.70	241.00 ± 13.23	233.33 ± 11.59	240.33 ± 13.61

Table S5: Concentrations of zinc in µg/L measured during the TEER measurements of WT and TAS2R43ko cells in the apical and basal compartment after the treatment with $ZnCl_2$ (0, 100, 500, and 1000 µM) for 30 min.

$ZnCl_2$	100 µM		500 µM		1000 µM	
	WT	TAS2R43ko	WT	TAS2R43ko	WT	TAS2R43ko
apical	5427.4	4173.4	23687.4	23141.0	35498.6	33199.1
basal	23.5	0.6	58.0	18	107.1	100.7
%	*0.4*	*0.0*	*0.2*	*0.1*	*0.3*	*0.3*

Table S6: Microarray screening for genes of zinc transporter-, calcium, and transmembrane-proteins in HGT-1 cells after 30 min exposure to 1000 µM $ZnCl_2$ in comparion to the untreated control cells showing beside the protein and its gene id the statistic, degree of freedom (*df*), *p*-value and effect size (effsize), summarized in its magnitude.

protein	gene	statistic	df	*p*	effsize	magnitude
**SLC30A1 (ZnT1)**	NM_021194	-1.71	2.06	0.23	-1.40	**large**
**SLC30A2 (ZnT2)**	NM_001004434	-1.14	3.75	0.32	-0.93	**large**
**SLC30A5 (ZnT5)**	NM_022902	-1.62	2.08	0.24	-1.32	**large**
**SLC39A2 (ZIP2)**	NM_014579	-1.72	2.28	0.21	-1.40	**large**
**SLC39A3 (ZIP3)**	NM_144564	2.68	3.50	0.06	2.19	**large**
**SLC39A11 (ZIP11)**	NM_139177	-1.65	2.20	0.23	-1.35	**large**
**SLC39A14 (ZIP14)**	NM_015359	-1.06	2.95	0.37	-0.87	**large**
**GPR39**	NM_001508	1.59	3.58	0.20	1.30	**large**
**S100G**	NM_004057	-1.16	3.73	0.32	-0.94	**large**
**S100A**	NM_006271	-4.14	3.36	0.02	-3.38	**large**
**ATP6V1E2**	BC008981	5.01	3.47	0.01	4.09	**large**
**ATP8B2**	NM_020452	4.08	2.80	0.03	3.33	**large**
**ATP8B5P**	BC031276	3.10	2.23	0.08	2.53	**large**
**CA5B**	AB021660	1.67	3.76	0.18	1.36	**large**
**CA5B**	NM_007220	1.65	2.35	0.22	1.34	**large**
**CA5BL**	XM_933655	3.66	2.12	0.06	2.99	**large**
**CA8**	NM_004056	1.93	3.01	0.15	1.58	**large**
**CCCTC zinc finger**	NM_080618	-2.83	3.48	0.06	-2.31	**large**
**RUVBL1**	NM_003707	2.16	3.96	0.10	1.77	**large**
SLC30A5 (ZnT5)	NM_024055	0.72	3.80	0.51	0.59	moderate
SLC30A6 (ZnT6)	NM_017964	0.86	2.97	0.46	0.70	moderate
SLC39A6 (ZIP6)	NM_012319	-0.72	2.96	0.53	-0.59	moderate
SLC39A8 (ZIP8)	NM_022154	0.82	2.17	0.49	0.67	moderate
SLC39A9 (ZIP9)	NM_018375	-0.65	3.51	0.56	-0.53	moderate
SLC30A3 (ZnT3)	NM_003459	0.02	3.99	0.99	0.02	negligible
SLC30A4 (ZnT4)	NM_013309	-0.22	3.78	0.84	-0.18	negligible
SLC30A8 (ZnT8)	NM_173851	-0.14	3.10	0.90	-0.11	negligible
SLC39A3 (ZIP3)	NM_213568	-0.11	3.97	0.92	-0.09	negligible
SLC39A4 (ZIP4)	NM_130849	-0.19	2.25	0.86	-0.16	negligible
SLC39A4 (ZIP4)	NM_017767	-0.22	2.45	0.84	-0.18	negligible
SLC39A10 (ZIP10)	NM_020342	0.09	3.06	0.94	0.07	negligible
SLC30A7 (ZnT7)	NM_133496	-0.43	3.16	0.69	-0.35	small
SLC30A9 (ZnT9)	NM_006345	0.44	3.59	0.68	0.36	small
SLC39A1 (ZIP1)	NM_014437	0.52	3.99	0.63	0.42	small
SLC39A5 (ZIP5)	NM_173596	0.41	2.59	0.72	0.33	small
SLC39A7 (ZIP7)	NM_006979	0.27	3.88	0.80	0.22	small
TMEM163	NM_030923	-0.31	2.01	0.78	-0.25	small
SLC39A12 (ZIP12)	NM_152725	0.35	2.17	0.76	0.28	small
SLC39A13 (ZIP13)	NM_152264	0.59	3.92	0.59	0.48	small
MT3	NM_005954	-0.39	3.02	0.72	-0.32	small
SLC1A1	NM_004170	-0.56	3.97	0.61	-0.46	small

Table S7: Intensities of the immunocytochemistry staining as corrected total cell fluorescence (CTCF) of ZnT1, ZnT5, ZIP4, and ZIP14 in HGT-1 WT and TAS2R43ko cells after incubation with $ZnCl_2$ for 30 min (100, 500, and 1000 µM). Intensity increase in TAS2R43ko cells calculated in %.

**ZIP4**	**0**	**100 µM**	**500 µM**	**1000 µM**	**P VALUE**
**WT**	17.28 ± 7.28	13.33 ± 5.35	15.40 ± 6.61	19.08 ± 7.86	**
**43KO**	16.24 ± 5.00	14.17 ± 2.27	20.78 ± 6.29	21.02 ± 8.26	****
**MEAN FC**	*0.9*	*1.1*	*1.3*	*1.1*	
	ns	*ns*	****	*ns*	
**ZIP14**					
**WT**	2.84 ± 0.94	1.84 ± 0.84	1.94 ± 0.38	1.36 ± 0.41	***
**43KO**	7.50 ± 1.74	2.61 ± 1.26	9.18 ± 2.27	15.10 ± 6.22	****
**FC**	*2.6*	*1.4*	*4.7*	*11.1*	
	ns	*ns*	****	****	
**ZNT-1**					
**WT**	1.29 ± 0.52	1.12 ± 0.28	0.79 ± 0.41	0.68 ± 0.16	**
**43KO**	1.58 ± 0.35	1.17 ± 0.56	1.44 ± 0.15	2.54 ± 1.18	****
**FC**	*1.2*	*1.0*	1.8	*3.7*	
	ns	*ns*	****	****	
**ZNT5**					
**WT**	18.44 ± 4.95	7.25 ± 3.03	6.16 ± 2.12	13.15 ± 6.78	****
**43KO**	9.71 ± 5.77	9.66 ± 3.46	12.42 ± 4.72	13.26 ± 3.21	***
**FC**	*0.5*	*1.3*	*2.0**	*1.0*	
	****	*	****	*ns*	

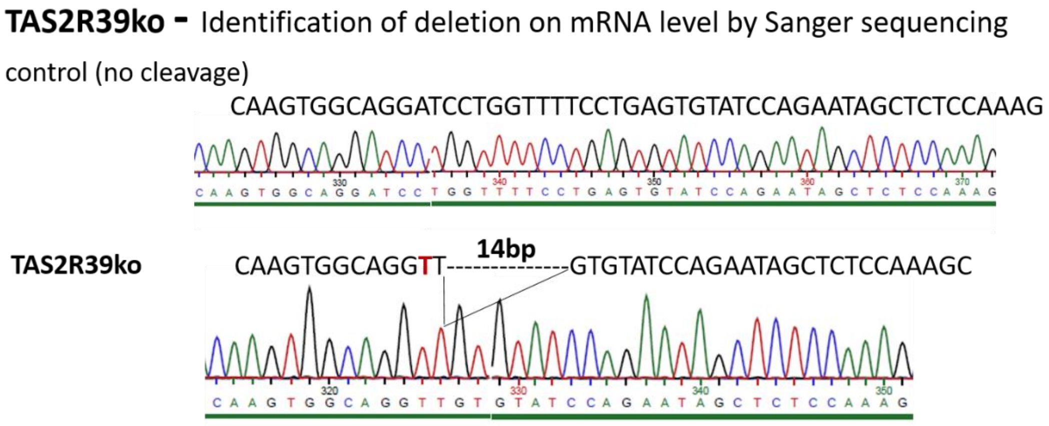

Figure S1: Verification of deletion on mRNA level by Sanger sequencing compared to WT HGT-1 cells.

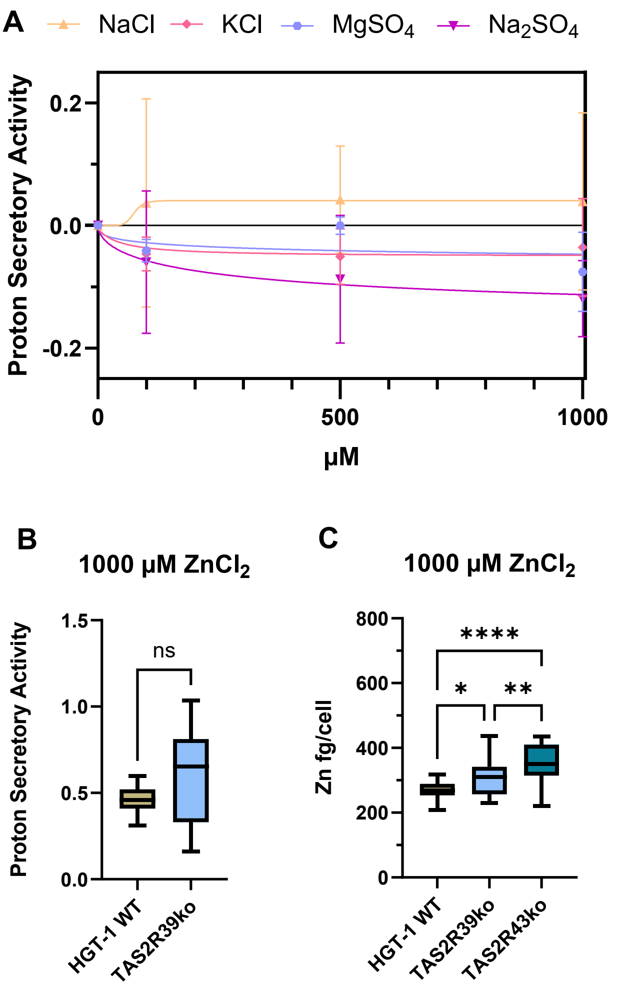

Figure S2:

(A) Secretory activity of HGT-1 cells 10 min after the treatment with metal salts $MgSO_4$, Na_2SO_4, KCl, and NaCl for 30 min at concentrations between 100 - 1000 µM displayed as mean ± SD, n = 3-6, t.r. = 3.

IPX of (B) HGT-1 WT and TAS2R39ko after treatment with 1000 µM ZnCl2 and (C) Cellular concentration of zinc in fg/cell when HGT-1 WT, TAS2R39ko and TAS2R43ko cells treated with ZnCl2 (100, 500, and 1000 µM). Comparison via ANOVA * = $p \leq 0.05$, ** = $p \leq 0.01$, **** = $p \leq 0.00001$.

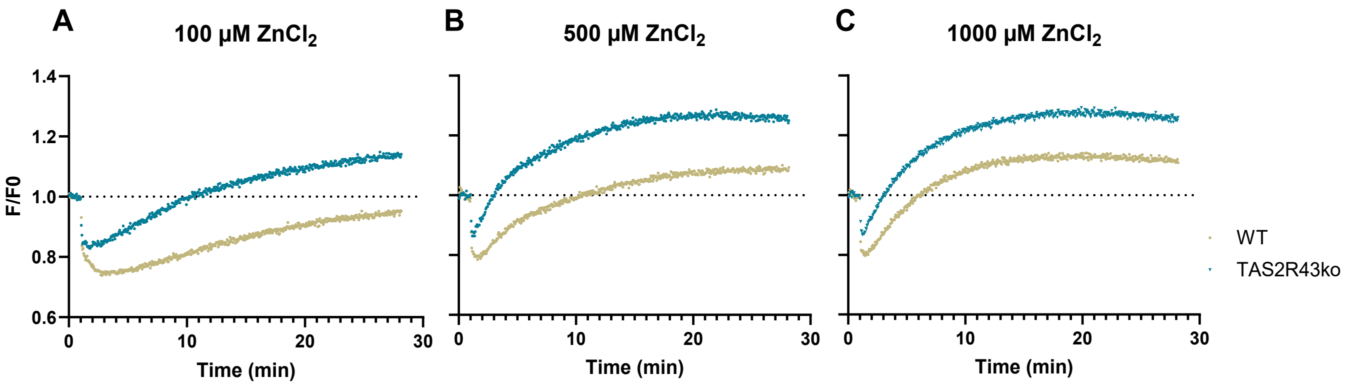

Figure S3: Ca^{2+} release in WT and TAS2R43ko cells injected with $ZnCl_2$ ((A) 100, (B) 500 and (C) 1000 µM) in C1 buffer 60 sec after starting the measurements. The dye itself reacted in the cell free experiment with 100, 500, and 1000 µM ZnCl2 approaching f/f0 values between 1.39 - 1.50, .32 - 1.41, and 1.22 - 1.30.

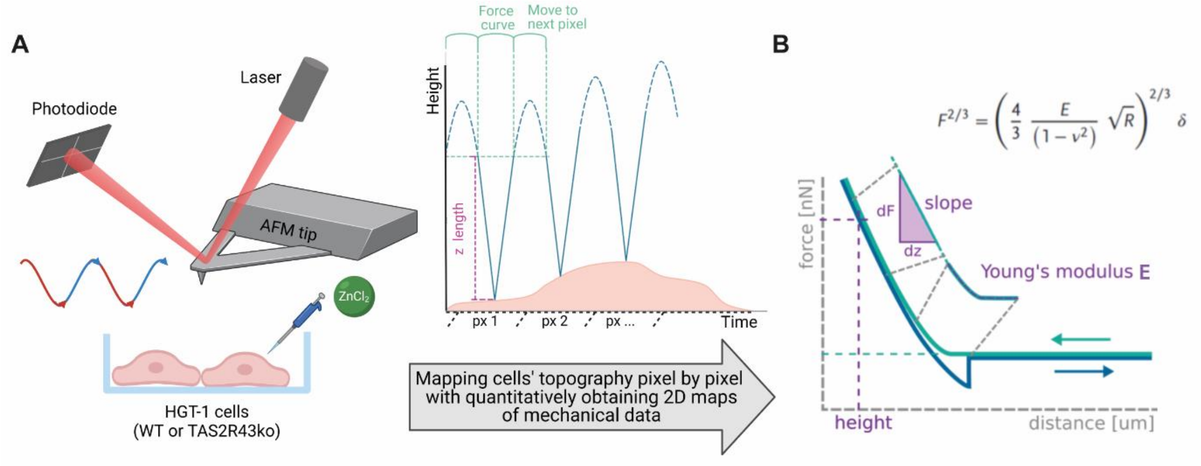

Figure S4: **(**A) AFM technique performed on HGT-1 cells describing (B) the ratio between stress and strain, as well as height.

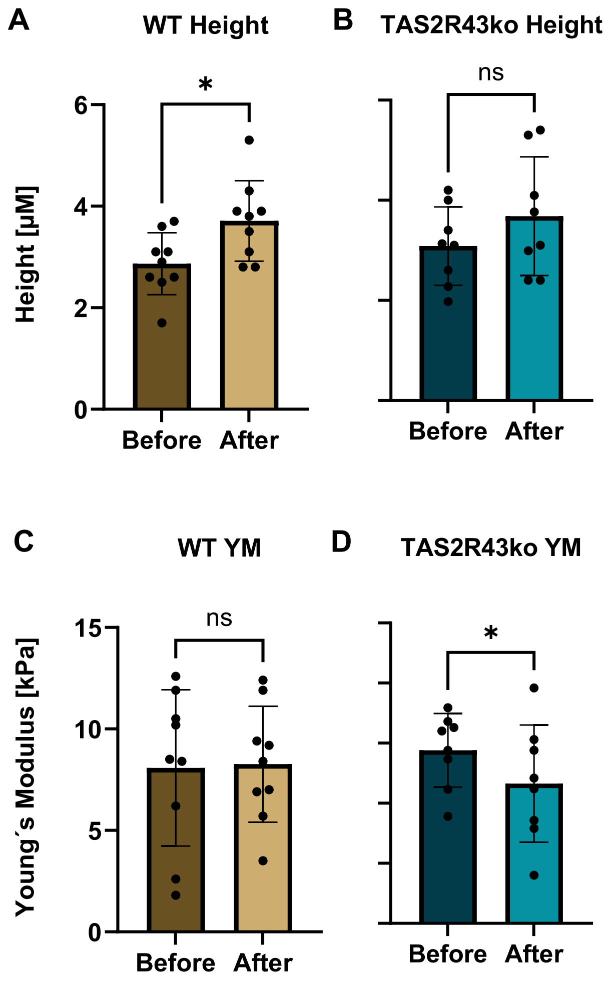

Figure S5: Height and Stiffness of WT (A, C) and TAS2R43ko (B, D) cells before and after the 30 min treatment with 1000 µM $ZnCl_2$.

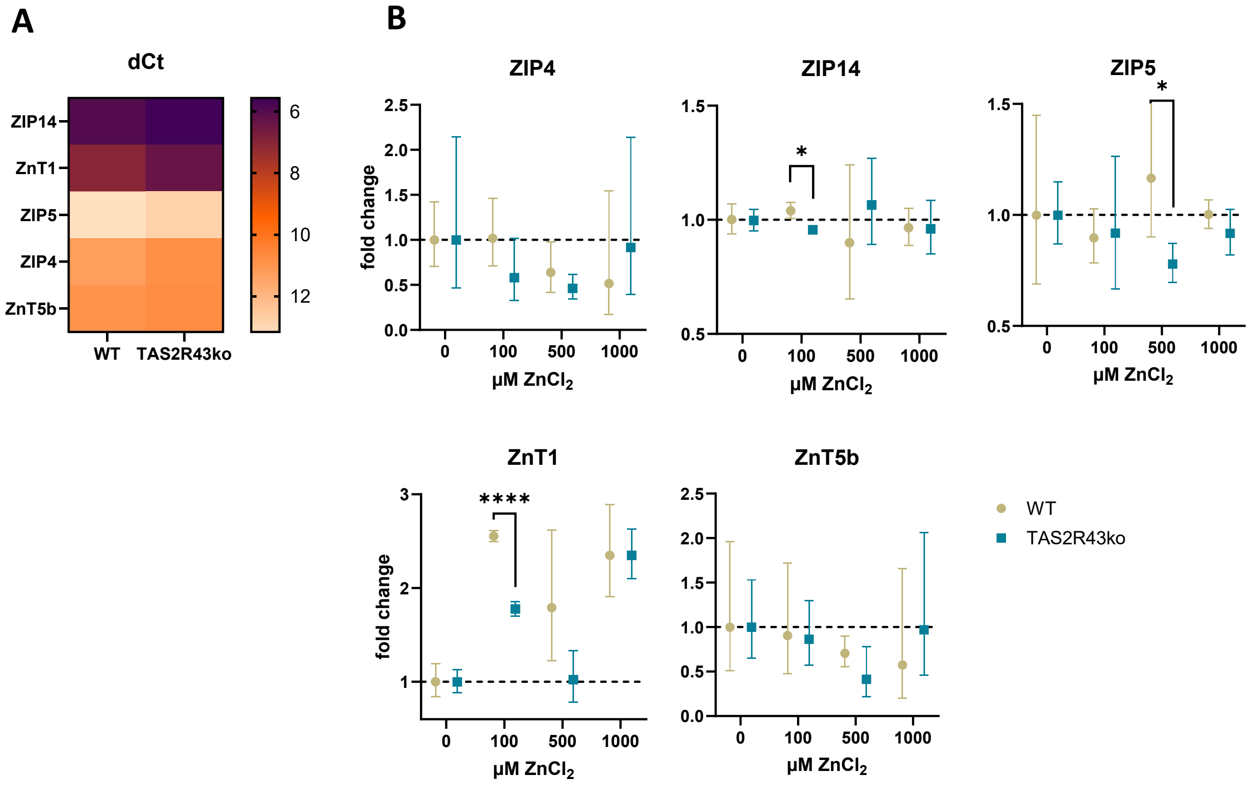

Figure S6:

(A) Heatmap displaying the mean dCt expression of the zinc transporter proteins ZIP14, ZnT1, ZIP5, ZIP4, and ZnT5b in untreated WT and TAS2R43ko cells.

(B) Fold change of ZIP14, ZnT1, ZIP5, ZIP4, and ZnT5b after exposure to ZnCl2 (untreated, 100, 500, and 1000 μM). Statistics by t-test between WT and TAS2R43ko cells * = $p \leq 0.05$, **** = $p \leq 0.0001$.

ZIP4

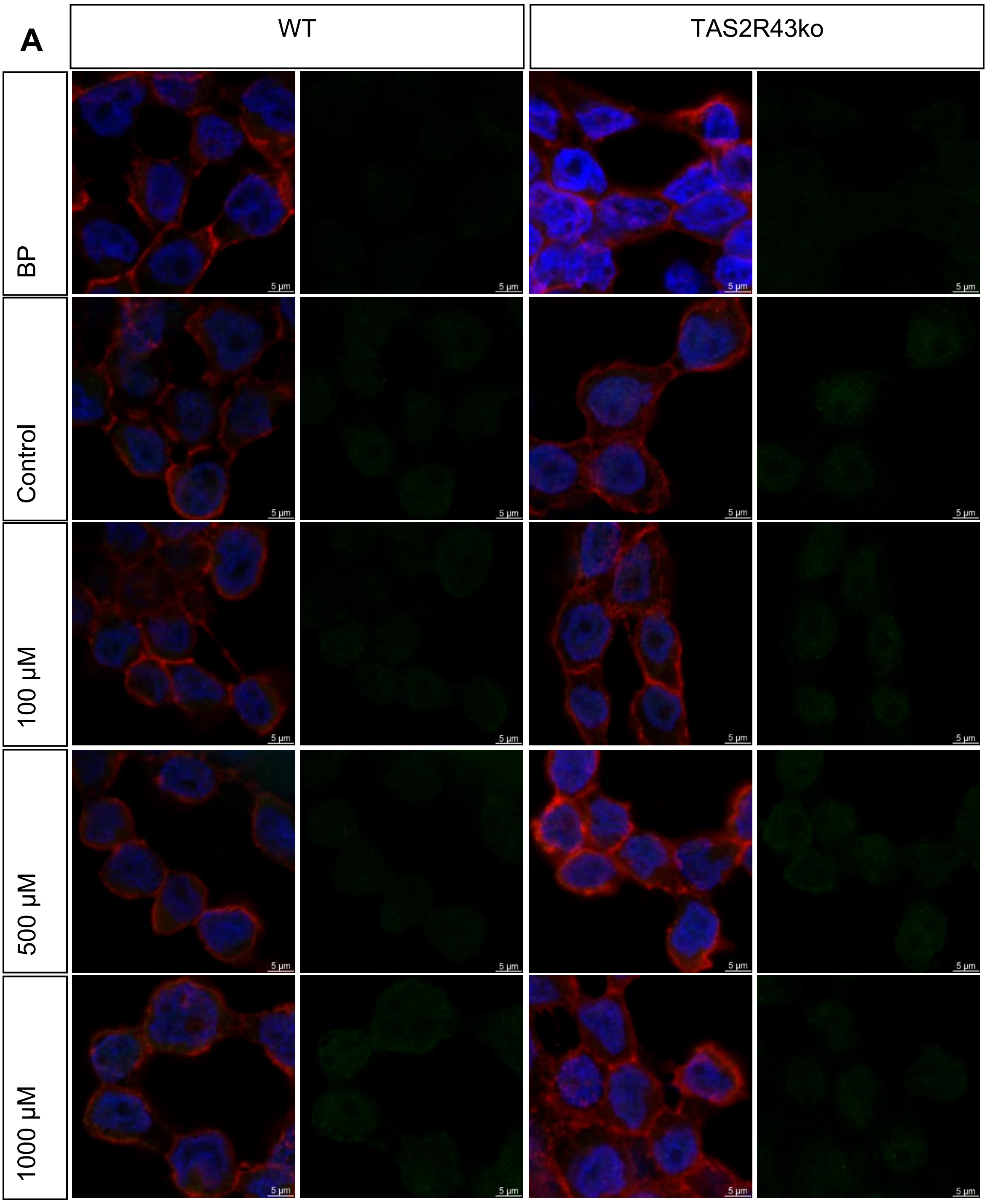

ZnT1

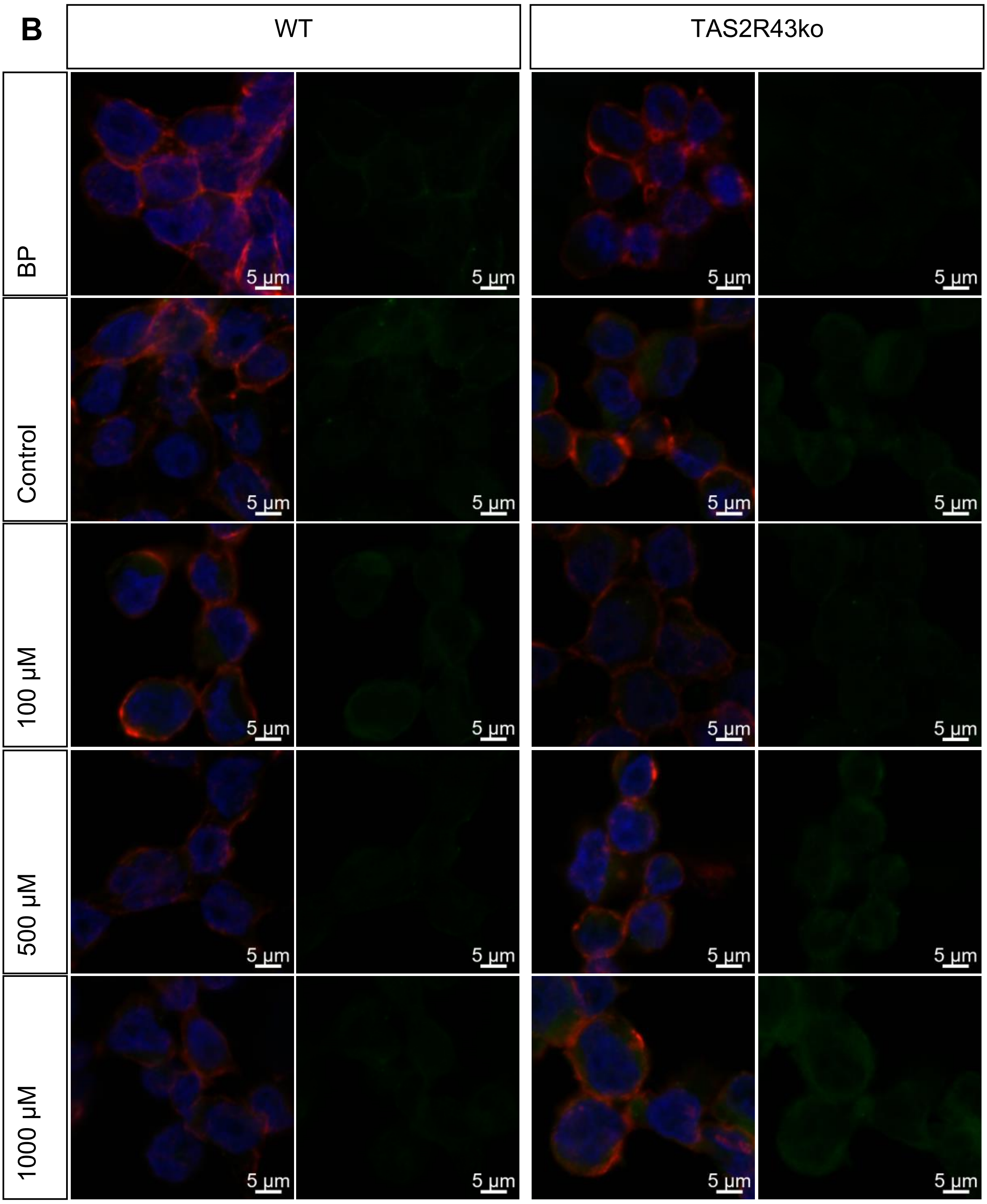

ZnT5b

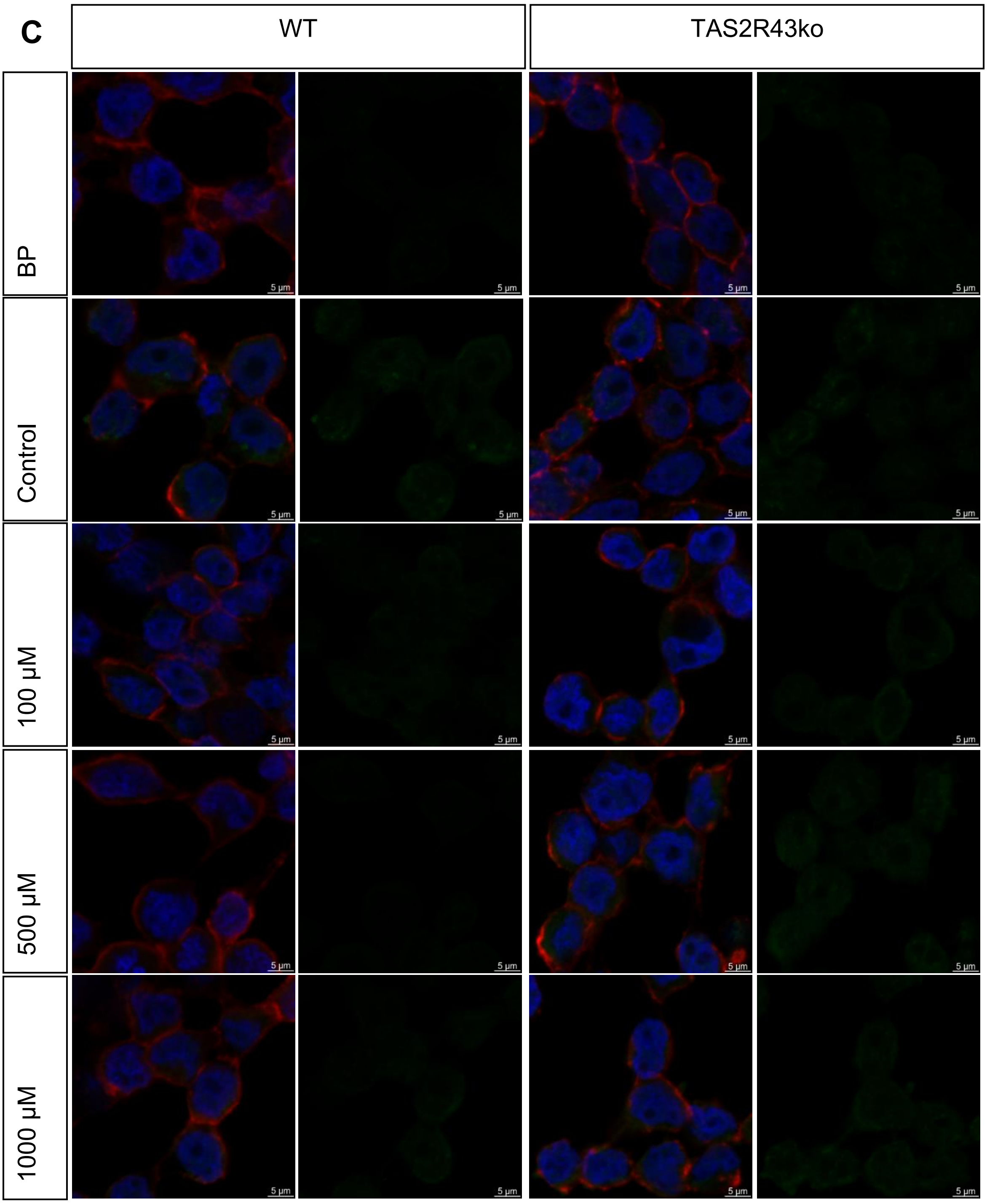

Figure S7: Immunocytochemistrystaining (ICC) (A) ZIP4, (B) ZnT1, and (C) ZnT5 in HGT-1 WT (left) and TAS2R43ko (right) cells as overlay and the channel of the Zn-transporter after the treatment with $ZnCl_2$ (100, 500, and 1000 µM) for 30 min prior the staining procedure. Fluorescence dyes: Nucleus in blue (Hoechst), cell membrane in red (Alexa 633), Zn-transporter in green (Alexa 488).

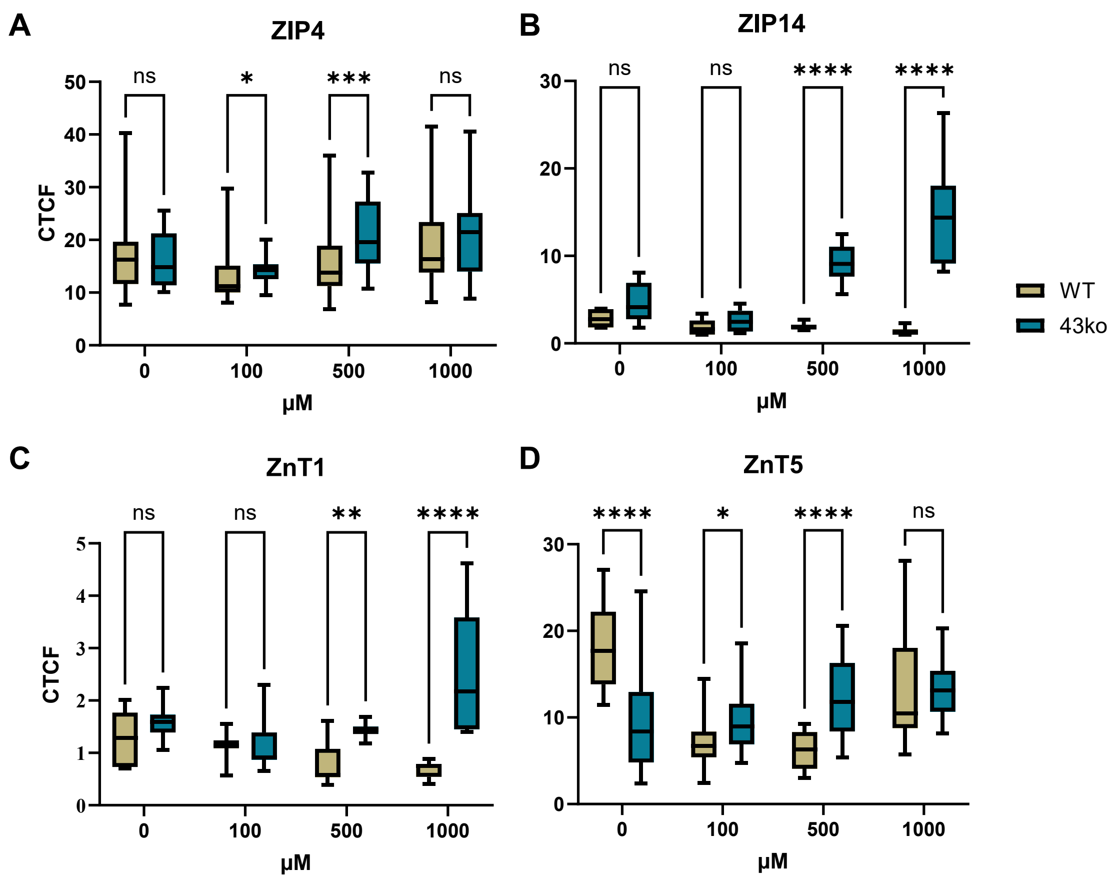

Figure S8:

CTCF of ZIP4 (A), ZIP14 (B), ZnT1 (C), and ZnT5 (D) on WT and TAS2R43ko cells after the treatment with $ZnCl_2$ as mean ± SD. Statistical difference between WT and TAS2R43ko cells tested by multiple Mann-Whitney tests and displayed as * = $p \leq 0.01$, **** = $p \leq 0.0001$.
